# DNA Methylation Analysis Reveals Distinct Patterns in Satellite Cell–Derived Myogenic Progenitor Cells of Subjects with Spastic Cerebral Palsy

**DOI:** 10.3390/jpm12121978

**Published:** 2022-11-30

**Authors:** Karyn G. Robinson, Adam G. Marsh, Stephanie K. Lee, Jonathan Hicks, Brigette Romero, Mona Batish, Erin L. Crowgey, M. Wade Shrader, Robert E. Akins

**Affiliations:** 1 Nemours Children’s Research, Nemours Children’s Health System, Wilmington, DE 19803, USA; 2 Center for Bioinformatics and Computational Biology, University of Delaware, Newark, DE 19716, USA; 3 Medical and Molecular Sciences, University of Delaware, Newark, DE 19716, USA; 4 Department of Orthopedics, Nemours Children’s Hospital Delaware, Wilmington, DE 19803, USA

**Keywords:** cerebral palsy, muscle spasticity, primary cell culture, satellite cells, skeletal muscle, muscle, skeletal, humans, epigenomics, DNA methylation, regulatory non-coding RNAs

## Abstract

Spastic type cerebral palsy (CP) is a complex neuromuscular disorder that involves altered skeletal muscle microanatomy and growth, but little is known about the mechanisms contributing to muscle pathophysiology and dysfunction. Traditional genomic approaches have provided limited insight regarding disease onset and severity, but recent epigenomic studies indicate that DNA methylation patterns can be altered in CP. Here, we examined whether a diagnosis of spastic CP is associated with intrinsic DNA methylation differences in myoblasts and myotubes derived from muscle resident stem cell populations (satellite cells; SCs). Twelve subjects were enrolled (6 CP; 6 control) with informed consent/assent. Skeletal muscle biopsies were obtained during orthopedic surgeries, and SCs were isolated and cultured to establish patient–specific myoblast cell lines capable of proliferation and differentiation in culture. DNA methylation analyses indicated significant differences at 525 individual CpG sites in proliferating SC–derived myoblasts (MB) and 1774 CpG sites in differentiating SC–derived myotubes (MT). Of these, 79 CpG sites were common in both culture types. The distribution of differentially methylated 1 Mbp chromosomal segments indicated distinct regional hypo– and hyper–methylation patterns, and significant enrichment of differentially methylated sites on chromosomes 12, 13, 14, 15, 18, and 20. Average methylation load across 2000 bp regions flanking transcriptional start sites was significantly different in 3 genes in MBs, and 10 genes in MTs. SC derived MBs isolated from study participants with spastic CP exhibited fundamental differences in DNA methylation compared to controls at multiple levels of organization that may reveal new targets for studies of mechanisms contributing to muscle dysregulation in spastic CP.

## 1. Introduction

Cerebral palsy (CP) is the most common cause of physical disability in childhood, with a prevalence of 2–4 per 1000 live births [1,2,3]. It is a heterogeneous set of movement disorders associated with a static encephalopathy that occurs during fetal development or early postnatal life [4]. There are three fundamental types of CP: ataxic, dyskinetic, and spastic [5], with spastic CP accounting for about 80% of cases [6]. Spastic CP is characterized by hypertonia, exaggerated reflexes, and poor muscle growth associated with progressive musculoskeletal deformities that often require surgical correction [5,6,7,8,9,10,11,12,13].

Individuals with spastic CP have difficulties with movement, movement control, and muscle function [2,3]. Ultrasound and MRI studies have demonstrated that subjects with spastic CP have decreased muscle length [14], cross–sectional area [15], and volume [16,17], leading to diminished force generation, reduced range of motion, and weakness [18,19,20,21]. Additionally, studies of muscle indicate that patients with CP exhibit increased sarcomere length [22], disorganized neuromuscular junctions [23,24,25], extracellular matrix abnormalities [22], tissue–level differences in gene expression profiles [26,27], and limited myogenic potential [28].

While all individuals with spastic CP have some movement dysfunction, there is a high degree of variability in phenotype between individuals. A more thorough understanding of the mechanisms associated with dysfunction in the peripheral neuromotor system is needed in order to develop more targeted and enhanced therapeutics addressing the major challenges facing individuals with spastic CP. Importantly, spastic CP is associated with alterations in the muscle–resident stem cell populations (satellite cells; SC) responsible for skeletal muscle growth and repair. Surgical patients with CP have a reduced SC population, which may account for aspects of their impaired muscle growth and decreased ability to strengthen muscle [28,29,30]. Isolated SC–derived myoblasts (MB) from CP donors appear to have altered phenotypes in culture compared to control MBs [28,31,32], and an RNASeq study from our group showed differential expression of mRNAs and miRNAs in spastic CP muscle [26]. These data suggest that MBs from CP patients may retain intrinsic differences through the cell isolation and culture process.

Genetic alterations may account for intrinsic differences in isolated MB populations. A number of potentially causative genetic variants have been identified in CP [33], but not all types of CP are easily detected or characterized by genomic data. Several rare copy number variants and mutations have been identified, but there is considerable genetic heterogeneity in patients with CP [33,34]. While some CP cases may be associated with certain types of genetic abnormality [35], the conventional view of CP remains that environmental factors affecting neuromotor maturation are responsible for most cases, especially among individuals with spastic CP [36,37].

Epigenetic modification may also account for some retained phenotypic differences in isolated MB behavior. In recent work, DNA methylation pattern differences were identified in peripheral blood cells from subjects with CP [38,39,40,41], and some early studies indicate that muscle in CP may be similarly altered, with differential DNA methylation in CP resulting in a decreased capacity for MBs to fuse and differentiate into MTs [32]. It has been demonstrated in several disease states that patterns of altered DNA methylation may uncover molecular etiologies and reveal potential therapeutic targets [42,43,44,45,46,47,48,49,50,51], and such may be the case in spastic CP as well [39]. DNA methylation changes may serve as markers for diagnosis, prognosis, tailoring the best treatment for a subclass of disease, monitoring treatment efficacy, and identifying genes to be examined for the development of genetically or epigenetically targeted therapies [52]. In the current study, DNA methylomes were analyzed to provide insight into individual CpG site differences and altered DNA methylation patterns in chromosomal segments and near transcription start site (TSS) in spastic CP SCs compared to controls.

## 2. Materials and Methods

### 2.1. Subject Enrollment

Six subjects with a diagnosis of spastic CP and 6 control subjects were enrolled in an IRB–approved study at Nemours Children’s Hospital, Delaware, after informed consent/assent. The control cohort comprised children with an idiopathic condition or an injury. Subjects with a chromosomal disorder, degenerative neurological disease, or muscular dystrophy were excluded.

### 2.2. Satellite Cell Isolation

Skeletal muscle biopsies collected during orthopedic surgeries were enzymatically digested and a double–immunomagnetic isolation approach was used to collect a population of mononuclear Cells positive for the surface markers neural cell adhesion molecule 1 and C–X–C motif chemokine receptor 4 (anti–NCAM1 and anti–CXCR4, both at 2.5 ng/mL, Miltenyi, San Diego, CA, USA) as previously described [26]. Previous studies have demonstrated that CXCR4 marks human SCs and that selection using the combination of NCAM1 (CD56) and CXCR4 more effectively removes non–satellite cells than using either marker alone [53,54]. This method resulted in a nearly pure SC population as verified by positive PAX7 immunofluorescence signal obtained after 24–48 h in culture (Anti–PAX7 from hybridoma cells deposited to the DSHB by Kawakami, A., Developmental Studies Hybridoma Bank, Iowa City, IA, USA). Cell populations that were at least 90% positive for PAX7 expression were utilized for experiments (Supplemental Table S1).

Cells were seeded at passage 3–5 (Supplemental Table S1) and proliferated in medium consisting of Zenbio Skeletal Muscle Growth Medium (Zenbio, Triangle Park, NC, USA) supplemented to a final concentration of 20% Qualified FBS (Thermo Fisher Scientific, Philadelphia, PA, USA), 4 g/L of D Glucose (Thermo Fisher Scientific, Philadelphia, PA, USA), 1 ng/mL of human bFGF (PeproTech, Rocky Hill, NJ, USA), and 1% penicillin–streptomycin, (Thermo Fisher Scientific, Philadelphia, PA, USA) was exchanged every other day until cells reached confluence. Proliferating MBs were collected at 50% confluence (2–5 days after seeding; Supplemental Table S1). For differentiation, cultures were switched to low–serum medium consisting of high glucose DMEM (Thermo Fisher Scientific, Philadelphia, PA, USA) supplemented with 2% horse serum (Thermo Fisher Scientific, Philadelphia, PA, USA), 2% human insulin (Sigma, St. Louis, MO, USA) and 1% penicillin–streptomycin upon reaching 90–100% confluence. Cells were differentiated for 24 **h** to initiate MB fusion into MTs [28] and collected.

### 2.3. DNA Extraction, Library Preparation, and Sequencing

Genomic DNA was isolated using Gentra Puregene kits (Qiagen, Germantown, MD, USA). A previously published DNA methylation assay [39,55,56] was utilized. Briefly, DNA libraries for next generation sequencing (NGS) were prepared by digesting genomic DNA with methyl–sensitive restriction endonuclease HpaII, which recognizes CCGG sites. A standard sequencing protocol was then performed including randomized shearing (Covaris, Woburn, MA, USA) and synthesis of a gDNA fragment library using Illumina TruSeq Nano library synthesis kits (San Diego, CA, USA). NGS was performed on an Illumina ×10 platform by Psomagen (Rockville, MD, USA). The protocol generated single end reads (150 bp) with >20× coverage of the regions captured. FASTQ data files were processed to calculate the probability of methylation at individual CpG sites through a commercial bioinformatics pipeline and software platform (Genome Profiling LLC, Newark, DE, USA). For convenience, the term “CpG” in this paper refers to “C(CpG)G” HpaII restriction sites.

### 2.4. Methylation Analysis

FASTQ files were aligned to human reference genome hg19 using BWA (Burrows–Wheeler Aligner algorithm [57]. To reduce false discovery associated with the inclusion of both male and female participants in the study, sites on the X and Y chromosomes were excluded from analyses. For each CpG per sample, a methylation score was calculated proportional to the probability that a specific CpG site was methylated. These scores were then compared between cohorts using a set of analytic modules from the R–packages edgeR [58,59] and limma [60] to compare pairwise for each CpG site. The response scale of the methylation data sets used here is within the operational boundaries of log–scaled gene expression data for which edgeR and limma were designed, and the well–developed false–discovery rate calculations in these R packages are ideal for methylation score data distributions [61,62].

Statistical significance at the level of individual CpG sites and was evaluated using a Likelihood Ratio Test with a one–way ANOVA contrast (LRT–ANOVA). Potentially informative CpG sites were selected for the PCA plots by filtering the LRT–ANOVA *p*-values with an appropriate cutoff (<0.01). Gene annotations were derived from the Ensembl GRCh37 database based on chromosomal locus (www.ensmbl.org; last accessed on 15 November 2022 for validation). Enhancer regions were derived from Ensembl’s GRCh37 BioMart tool. The ontology terms Muscle Organ Development, Muscle System Processes, and Skeletal Muscle Cell Differentiation (GO Enrichment Analysis; amigo.geneontology.org) were used to determine 575 unique genes annotated to be involved in muscle physiology. Fisher’s exact test was utilized to determine enrichment of significant CpGs within chromosomes.

Because methylation of the region around the TSS of a gene is thought to be highly informative of gene expression [63], the individual CpG methylation scores were averaged per TSS using 1000 bp upstream of the TSS and 1000 bp downstream [64]. TSS were identified from Ensembl’s GRCh37 BioMart data mining tool (release 106) [65]. If one or fewer CpGs were found within this range, the TSS was excluded from further analysis. For each sample, the mean methylation load in this 2000 bp range was calculated and a likelihood ratio test performed on the methylation loads. No more than one transcript for each gene was included in the statistical analysis. 

## 3. Results

Samples from 12 unique study participants were included in the study; demographic data are summarized in Table 1. Briefly, the control group consisted of *n* = 6 subjects (males = 3, and females = 3) with an average age of 13.9 ± 1.7 years; the CP group consisted of *n* = 6 subjects (males = 3, and females = 3) with an average age of 15.5 ± 3.0 years. Biopsies from different muscles were included based on the availability of viable muscle tissue suitable for cell isolation and to help identify methylation signals generally associated with spastic CP rather than a specific muscle: SCs were isolated from spinalis or semitendinosus muscle for controls and from spinalis, rectus femoris, adductor longus, or vastus lateralis for the CP group. Although differences likely exist between muscle types, previous studies of MTs derived from different muscles found low inter-muscle variability in RNA–sequencing data [26].

**Figure 1:**
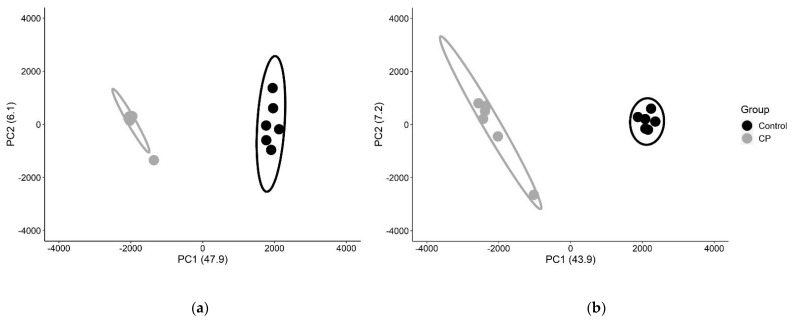
Principal component analysis to identify discriminating methylation patterns between CP and non–CP cohorts. For MBs (**a**) and MTs (**b**), the first two component axes (PC1, PC2) were plotted with % variance explained in parenthesis. Each point represents the similarity position of a subject based on all potentially informative CpG sites (*p* < 0.01). CP subjects are represented in gray and control subjects in black. Ellipses represent 90% confidence intervals. The complete segregation of the two cohorts indicates that DNA methylation patterns fundamentally differ between cohorts.

To evaluate differences in DNA methylation patterns associated with affected muscle of subjects with spastic CP, whole genome methylation patterns were determined from proliferating MBs and differentiating MTs. Cells derived from subjects with CP appeared morphologically similar to those derived from controls (Supplemental Figure S1) and exhibited no significant differences in proliferation rates (Supplemental Table S1). NGS was performed after methylation sensitive restriction endonuclease (HpaII) digestion. The hg19 reference genome assembly from the University of California Santa Cruz [66] includes 2.29 × 10^6^ HpaII target CCGG motifs, which represent ~15% of the 14 × 10^6^ CpG sites in the haploid hg19 genome [39]. Alignment of the HpaII restricted sites in our 12 samples yielded 1,483,038 sites were in common across all subjects for MBs and MTs. DNA methylation patterns were analyzed at the individual CpG site level using dimensionality reduction by principal component analysis (PCA) to assess the degree of discrimination between CP and non–CP cohorts. All potentially informative CpG sites (*n* = 20,254 for MBs and 27,834 for MTs), were integrated as one pattern and demonstrated strong discrimination based on diagnosis (Figure 1, Supplemental Figure S2).

**Figure 2:**
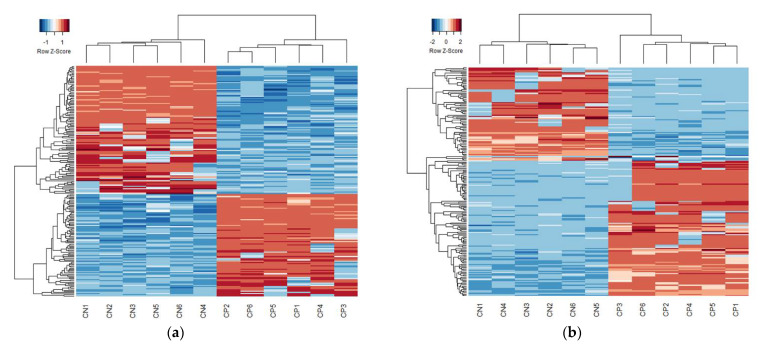
Heatmap clustering of the top 200 CpG sites. Of the common CpG sites, there was significant differential methylation in 525 distinct CpGs for MBs (**a**) and 1774 for MTs (**b**) (FDR–corrected *p*-value < 0.05). Heatmaps based on the 200 CpG sites with the lowest FDR—corrected *p*-values were generated using Euclidean distances and complete linkage clustering. Each row represents the score for a single CpG site across all subjects with blue indicating hypomethylation and red indicating hypermethylation. Quantitative differences in CpG site methylation by diagnosis were apparent.

In MBs, 525 CpG sites were found to have differential methylation load scores (FDR < 0.05; heatmap in Figure 2a, volcano plot in Supplemental Figure S3A, list of significant CpGs in Supplemental Table S2). Of these, 11 were within genes known to be involved in muscle physiology and 21 were within known gene enhancer regions. 1774 CpG sites were found to have differential methylation load scores in MTs between the CP and control cohorts (heatmap in Figure 2b, volcano plot in Supplemental Figure S3B, list of significant CpGs in Supplemental Table S3), of which 43 CpG sites were within genes known to be involved in muscle physiology and 97 were within gene enhancer regions. The differentially methylated CpGs included 79 CpG sites that were significantly different under both cell conditions. Of these, 36 were significantly hypermethylated and 43 were significantly hypomethylated in the CP cohort compared to the control cohort (Table 2).

**Figure 3:**
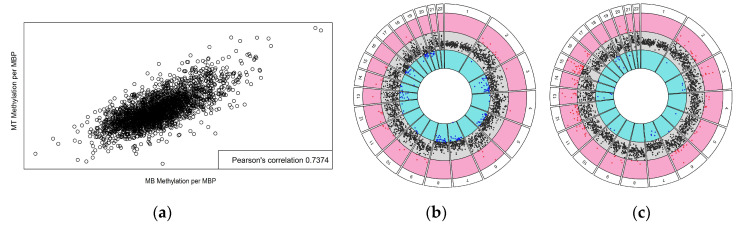
Chromosome–based circos plots. Mean logFC values for CpG methylation load (CP over control) were calculated for sequential 1 Mbp intervals. There was a strong correlation between differential methylation in MBs and MTs (**a**). Scatterplots of values for each chromosome are shown for MBs (**b**) and MTs (**c**); values outside the 95% confidence interval of the average logFCs across the whole genome are red indicating significant hypermethylation in CP or blue for significant hypomethylation in CP. The gray ring shows points within the 95% CI for the overall data.

To determine the methylation differences over larger regions of the genome, the chromosomal distribution of significant CpGs was visualized (Supplemental Figure S4) and Fisher’s exact test was employed to analyze enrichment of differentially methylated CpGs on individual chromosomes. For both MB and MT cell populations, significant enrichment was found on chromosomes 12, 13, 14, 15, 18, and 20. Interestingly, the same chromosomes were identified as having significant CpG site enrichment in previous studies of both muscle tissue and peripheral blood cells from subjects with CP (Table 3). To further assess regional differences, methylation load scores were calculated across 1 Mbp chromosomal segments. There was a strong correlation between the 1 Mbp methylation loads of MBs and MTs (Figure 3a), indicating stability of the methylome. When these 1 Mpb regions were mapped to the chromosomes, regions of accentuated differential methylation load were noted on all chromosomes except 1 and 17 (Figure 3b,c). These large–scale changes in DNA methylation could affect higher–order chromatin structure and regulation of gene expression [52]. 

Since DNA methylation in promoter regions of genes has been associated with regulation of expression, the individual CpG methylation scores were averaged across TSS flanking regions using 1000 bp upstream of the TSS and 1000 bp downstream. The promoter methylation data revealed distinct patterns between the control and CP cohorts for both MBs (Table 4) and MTs (Table 5). Of 31,844 unique promoters identified, there were 3 promoters with statistically different methylation loads between CP and control subjects in the MBs and 10 promoters in MTs (FDR < 0.05). The majority of the differentially methylated promoters were in non-coding genes, with only one of the MB promoters and two of the MT promoters associated with protein coding transcripts. To explore the relationship between methylation loads and RNA expression, the correlation of methylation load in protein coding genes was analyzed against previously published RNA–seq count data per gene, but no correlation was found (data not shown).

## 4. Discussion

This study found that DNA methylation patterns in skeletal muscle SCs grown in culture differed significantly between a cohort of study participants with spastic CP and non–CP controls. DNA methylation is a common and widespread chemical modification involving the addition of a methyl group to the 5–carbon position of cytosine, predominantly within CpG dinucleotides [67]. DNA methylation patterns can change during normal developmental processes, and it has been shown that altered DNA methylation can be passed to daughter cells and sustained later in life [68,69,70,71,72,73,74,75]. Specific DNA methylation changes can modify gene expression, and DNA methylation is well known to be involved in X–chromosome inactivation, gene imprinting, and the silencing of transposable elements [76]. Changes in DNA methylation patterns can also occur as a result of pathophysiologic processes or acute exposures to environmental or physiologic stress [77,78,79]. Altered DNA methylation has been linked to a number of risk factors and potential causes for CP including prematurity, hypoxia–ischemia, and infection [80,81,82,83]. In a prior study, we found that DNA methylation patterns in peripheral blood cells of spastic CP patients varied significantly from controls [39], raising the possibility of methylome alterations in both hematopoietic stem cell and myogenic stem/SC lineages in spastic CP. Furthermore, epigenetic patterns from adolescents were able to be used to predict diagnosis of much younger patients [39], suggesting that at least some methylation pattern differences are associated with the onset of CP and are preserved over time.

The present study examined methylation pattern differences but did not look directly at differences in cell phenotype or behavior. Studies show that individuals with CP have significantly reduced numbers of SCs and that MBs derived from these SCs have a decreased capacity to fuse and differentiate into MTs in culture [29,84]. A recent report indicated that SC–derived MB progenitors from contractured muscle in CP have globally hypermethylated DNA and gene expression patterns that favor proliferation over quiescence and differentiation; in that study, a 24 h treatment with a hypomethylating agent reduced DNA methylation to control levels and promoted an exit from mitosis [32]. While previous studies of DNA methylation in CP SC–derived MBs have either examined DNA methylation of a specific CpG island [28] or used Infinium Human MethylationEPIC Beadchip arrays of 850,000 targeted CpGs to identify hypermethylated regions [32], the current study used a different sequencing technology and computational pipeline to examine over 1.4 million CpGs distributed throughout the genome in both MBs and MTs. In addition, the current study was able to take advantage of more closely age–matched samples than had been possible previously. This combination of more closely matched controls and a broader technology platform allowed for identification of both hypermethylated and hypomethylated regions as well as individual CpGs, which provide higher likelihood candidates for biomarker platforms.

The identification of differentially methylated CpG sites and regions in common across cells from the CP cohort suggests that fundamental molecular alterations associated with diagnosis were sustained after the cells were removed from the structural, biomechanical, and humoral environments of the muscle tissue. Such an effect has been described as muscle epigenetic memory wherein DNA methylation is stably altered by prior events like biomechanical loading or acute early life exposure to inflammatory cytokines [85]. Here, we identified 525 CpGs in MBs and 1774 in MTs that were differentially methylated in spastic CP versus controls. Interestingly, MBs demonstrated similar proliferation rates and RNA–seq profiles between cohorts in a previous study [26], indicating that while these 525 CpGs may be biomarkers for CP, they may not be associated with functional changes within the cells. Further work is needed to elucidate fully specific linkages between DNA methylation and the regulation of protein levels and cell activities. The larger number of significant sites in MTs was consistent with a larger number of differentially expressed genes in RNA–seq and may indicate that a mixture of cells at different stages of myogenic differentiation was present at the time of DNA isolation. In addition, reports indicate that native SC populations actually comprise multiple different subpopulations that may differentially contribute to variability; studies focused on clonal cells rather than heterogenous populations may be needed. Interestingly, in both MBs and MTs, several significant CpG sites were within genes known to be involved in muscle physiology, including skeletal muscle differentiation (HLF, NOTCH1), muscle organ development (BMP2, COL6A3, DCN, FZD2, HEG1, HLF, ITGA11, ITGA7, LAMA5, LARGE1, MAPK14, MYBPC1, MYH6, MYLK, NOTCH1, NRG1, PKP2, SGCD, SMAD7, TBX1, TCF12, TEAD4, WNT5A, ZFHX3, ZNF609), and muscle system process (ACTN3, ATP8A2, CACNA1C, CACNA1D, DTNA, DYSF, EDN3, GNAO1, HCN4, ITGB5, KCNQ1, LTB4R, MYBPC1, MYH6, MYLK, NEDD4L, PDLIM5, PKP2, PLA2G6, ROCK1, SGCD, TRDN). Of the differentially methylated CpGs identified in MBs and MTs, 79 were in common, with 36 being significantly hypermethylated and 43 significantly hypomethylated in the CP cohort under both conditions (Table 2). Alterations in DNA methylation can be sustained long–term and previous studies indicated that the majority of the DNA methylome remained relatively preserved through myogenesis, from SC to MT formation [76,86]. These 79 sites may therefore represent stable methylation signals indicative of CP; however, more studies are needed to determine the implication(s) of these differentially methylated sites and their roles in muscle impairment in CP.

Chromosome enrichment analysis determined that there was an enrichment of significant CpGs on chromosomes 12, 13, 14, 15, 18, and 20 for both MBs and MTs (Table 3). Interestingly, significant CpGs were also enriched on the same chromosomes in the CP cohort in skeletal muscle tissue and blood cells. An analysis of differential methylation over 1 Mpb regions in MBs demonstrated that chromosomes 6, 9, 11, and 21 contained regions of hypermethylation in the CP cohort, while chromosomes 5, 7, 8, 16, 19, and 20 contained regions of hypomethylation, and chromosomes 2, 3, 4, 10, 12, 13, 14, 15, and 18 contained regions of both hypermethylation and hypomethylation. MTs contained more hypermethylated regions and fewer hypomethylated regions in the CP cohort than MBs. Chromosomes 4, 8, 9, 11, 12, 14, 16, 21, and 22 contained regions of hypermethylation, while chromosome 7 contained regions of hypomethylation, and chromosomes 2, 3, 5, 6, 10, 13, 15, 18, and 20 contained regions of both hypermethylation and hypomethylation (Figure 3). Overall, the differential methylation levels over 1 Mbp regions were well correlated between MBs and MTs, again suggesting stability of the methylome during the course of myogenesis. The large regions of differential methylation between the CP and control cohorts throughout the genome suggest differences in chromatin structure within the CP cohort as various chromatin states based on histone modifications and nucleosome positioning can determine DNA methylation patterning [67]. There are complex mechanisms underlying the molecular crosstalk between DNA and histone methylation [87], and additional studies are needed to investigate specific histone modifications in CP to understand this complex relationship. While we are in the early stages of unraveling how these alterations are relevant to CP, several key chromosomes were identified as potential targets for future investigation in spastic CP.

We also identified statistically significant differences in methylation of the promoter regions of genes and assessed the relationship between these differences and an RNA–seq study of the same samples [26] to investigate the effect of differential DNA methylation on gene expression. Of particular note, specific associations between protein coding DNA methylation patterns and RNA expression were not readily resolved in our study. However, methylation/expression relationships are difficult to resolve in general and several studies have demonstrated that relationships between methylation status and gene expression can be complex [88,89]. Additionally, there is no current gold standard method for rolling up methylation scores across groups of individual CpG sites into a relevant burden for individual genes. Furthermore, approximately 95% of CpG island promoter regions are unmethylated independent of gene activity and recent studies suggest that methylation of promoter CpG islands is not the primary determinant of gene activity [52,90]. Many CpG islands occur in gene bodies, intergenic regions, or enhancers and may be relevant to gene expression [52]. In fact, recent studies have suggested that altered methylation in enhancer regions rather than promoter regions may be more indicative of changes in gene expression [52,91,92,93]. Enhancers can regulate the transcription of one or more genes, regardless of orientation or relative distance to the target promoter [94]. While 21 differentially methylated CpGs were identified within annotated enhancer regions in MBs and 97 in MTs, enhancers are difficult to map experimentally [52], enhancer activity is context and stimulus–dependent, and there are no genome–wide enhancer sets linked to specific promoters [94]. It was not possible to investigate the effects on gene expression due to these limitations in the annotation of enhancer elements. Therefore, it will be essential to continue investigating genome–wide analysis approaches that can accurately associate high–throughput expression data with methylation signatures.

Of note, the majority of the differentially methylated promotors identified in MBs and MTs were for regulatory RNAs; a result that may be indicative of differences in RNA processing within cells isolated from CP tissue (Table 4 and Table 5). These regulatory RNAs comprise the majority of the transcriptome and play critical roles in maintaining gene expression regulation. The most well–known regulatory RNAs include micro RNAs and long non-coding RNAs. MicroRNAs (miRNAs) are small noncoding RNAs (~22 nucleotides in length) that play important roles in developmental processes such as myogenesis and neurogenesis [95]. Long non-coding RNAs (lncRNAs) may mediate chromatin remodeling and modification, interact with transcription factors for gene regulation, interact with mRNAs to regulate post–transcriptional processes [96], and interact with miRNAs to facilitate myogenesis [97]. Additional studies are warranted to investigate the complex interplay between DNA methylation, histone modifiers, and non-coding RNAs in order to provide a comprehensive understanding of these epigenetic modulations on SC physiology and myogenesis in spastic CP.

The findings of our study compellingly support the idea that spastic CP is associated with altered epigenetic pathways, but our studies are limited by our reliance on the ability to obtain biopsies from individuals presenting for surgery. Because of this, our number of samples is small, SCs must be derived from different muscles in order to age–match samples, and our CP population presenting for surgery consists of mostly severely affected individuals with a high level of motor impairment and inactivity (GMFCS V); therefore, extrapolation of our results to a larger CP community may require additional research. Additionally, the technology used interrogates 2.29 × 10^6^ CCGG motifs, which represent ~15% of the 14 × 10^6^ CpG sites in the genome. The majority of the CpG sites in our study do not overlap with those selected for inclusion in the Infinium MethylationEPIC technology making comparisons to other studies using Infinium data challenging.

## 5. Conclusions

In this report, an innovative DNA methylation analysis was employed on SC–derived MBs and MTs collected from individuals with and without CP. We identified differential methylation in the CP cohort at the levels of individual CpGs, 1 Mpb regions, and promoters. The work presented here leverages our novel methylation approach with ex vivo cell studies to elucidate aberrant methylation signatures.

## Figures and Tables

**Table 1:** Demographic information for subjects in the study *.

Sample	Diagnosis	Age	Sex	GMFCS	Tissue Source
CN1	Spondylolysis	16.6	M	N/A	Spinalis
CN2	Torn ACL	12.6	M	N/A	Semitendinosus
CN3	Idiopathic scoliosis	12.1	F	N/A	Spinalis
CN4	Torn ACL	12.7	F	N/A	Semitendinosus
CN5	Idiopathic scoliosis	15.1	M	N/A	Spinalis
CN6	Idiopathic scoliosis	14.3	F	N/A	Spinalis
CP1	Spastic CP	15.6	M	5	Vastus lateralis
CP2	Spastic CP	19.1	M	5	Adductor longus
CP3	Spastic CP	12.6	M	4	Rectus femoris
CP4	Spastic CP	13.8	F	2	Rectus femoris
CP5	Spastic CP	19.0	F	5	Spinalis
CP6	Spastic CP	12.8	F	5	Spinalis

* CN = control; CP = cerebral palsy; ACL = anterior cruciate ligament; M = male; F = female; GMFCS = Gross Motor Function Classification System.

**Table 2:** CpG sites that were differentially methylated in both MBs and MTs *.

Position	MB LogFC	MB FDR Corrected *p*-Value	MT LogFC	MT FDR Corrected *p*-Value	Gene
chr2.0003882321	0.88	2.29 × 10^−2^	0.90	2.40 × 10^−2^	
chr2.0029850455	1.24	6.74 × 10^−4^	1.32	1.11 × 10^−5^	ALK
chr2.0033057636	−0.84	3.40 × 10^−3^	−0.85	1.94 × 10^−2^	LINC00486
chr2.0035092870	−1.09	1.00 × 10^−2^	−1.19	8.26 × 10^−5^	AC012593.1
chr2.0056193463	1.32	1.79 × 10^−3^	1.55	1.22 × 10^−6^	RP11—481J13.1, AC011306.2
chr2.0223166989	0.87	4.07 × 10^−2^	0.90	4.80 × 10^−2^	CCDC140
chr2.0235215325	−1.05	6.08 × 10^−4^	−1.08	6.49 × 10^−4^	
chr3.0053784559	0.91	1.58 × 10^−2^	1.14	8.96 × 10^−4^	CACNA1D
chr3.0060919598	−0.85	1.89 × 10^−3^	−0.86	3.09 × 10^−2^	FHIT
chr3.0119863345	1.28	2.04 × 10^−3^	1.48	1.19 × 10^−5^	GPR156
chr3.0119990864	−1.14	2.05 × 10^−3^	−1.01	4.01 × 10^−2^	GPR156
chr3.0127606140	−1.40	1.26 × 10^−4^	−1.12	6.36 × 10^−3^	
chr3.0182124231	−1.14	3.82 × 10^−2^	−1.15	5.10 × 10^−3^	
chr3.0189791239	−1.34	1.56 × 10^−2^	−1.13	5.00 × 10^−2^	LEPREL1
chr3.0196595774	−1.37	3.54 × 10^−2^	−1.33	1.44 × 10^−2^	SENP5
chr4.0101719592	−1.04	4.74 × 10^−5^	−1.15	1.77 × 10^−7^	EMCN
chr5.0011534641	0.90	2.48 × 10^−2^	1.05	7.82 × 10^−3^	CTNND2
chr5.0039219698	1.22	3.91 × 10^−2^	1.37	2.24 × 10^−2^	FYB
chr5.0164483805	−0.92	2.51 × 10^−2^	−1.11	1.05 × 10^−3^	CTC—340A15.2
chr5.0166472226	−1.03	1.03 × 10^−2^	−1.16	4.86 × 10^−4^	
chr6.0008948266	1.16	2.27 × 10^−2^	1.24	2.52 × 10^−3^	
chr6.0016145414	−1.17	2.94 × 10^−4^	−1.24	9.54 × 10^−5^	MYLIP
chr6.0019413218	0.81	2.95 × 10^−3^	0.86	1.70 × 10^−2^	
chr6.0031008851	0.96	2.29 × 10^−2^	1.01	1.56 × 10^−2^	RASSF3
chr6.0154640863	1.37	9.68 × 10^−3^	1.34	3.64 × 10^−3^	IPCEF1
chr6.0161063597	−2.29	3.71 × 10^−3^	−1.62	2.31 × 10^−2^	LPA
chr7.0016768868	−0.75	1.18 × 10^−2^	−1.03	1.03 × 10^−5^	
chr7.0044621160	0.91	1.71 × 10^−2^	0.97	4.26 × 10^−2^	TMED4
chr7.0147581299	−0.68	3.30 × 10^−2^	−0.83	3.24 × 10^−2^	CNTNAP2
chr11.0123045794	−1.36	1.13 × 10^−3^	−1.54	2.08 × 10^−6^	CLMP
chr11.0129565594	1.28	1.59 × 10^−2^	1.68	1.23 × 10^−4^	
chr12.0003241735	1.19	4.26 × 10^−3^	1.04	3.25 × 10^−2^	TSPAN9
chr12.0026672531	1.00	4.67 × 10^−2^	1.28	1.68 × 10^−2^	ITPR2
chr12.0048360477	−1.69	3.13 × 10^−4^	−1.10	4.25 × 10^−2^	TMEM106C
chr12.0054366343	0.87	4.94 × 10^−2^	1.07	3.53 × 10^−2^	HOTAIR
chr12.0055783991	1.19	4.36 × 10^−2^	1.29	2.11 × 10^−2^	
chr12.0083436417	1.62	4.94 × 10^−3^	2.14	6.72 × 10^−5^	TMTC2
chr12.0114887843	1.42	1.84 × 10^−4^	0.62	4.91 × 10^−2^	
chr12.0116068191	−1.32	1.42 × 10^−2^	−1.58	5.07 × 10^−6^	RP11—1028N23.4
chr12.0128167651	1.13	3.28 × 10^−2^	1.57	8.87 × 1014	
chr12.0131689822	1.29	2.19 × 10^−2^	1.74	5.09 × 10^−5^	RP11—638F5.1
chr13.0021286449	−1.10	4.87 × 10^−2^	−1.33	2.22 × 10^−2^	IL17D
chr13.0027424109	1.51	1.23 × 10^−2^	1.56	5.47 × 10^−4^	
chr13.0033220266	−1.24	5.87 × 10^−3^	−1.31	2.18 × 10^−3^	PDS5B
chr13.0047191668	−1.30	6.51 × 10^−3^	−1.34	6.36 × 10^−3^	LRCH1
chr13.0093896533	1.50	2.96 × 10^−2^	2.41	2.86 × 10^−5^	GPC6
chr13.0099687193	1.01	3.54 × 10^−2^	1.06	4.85 × 10^−2^	DOCK9
chr13.0107176083	−1.69	7.74 × 10^−3^	−1.68	1.29 × 10^−2^	EFNB2
chr13.0109856377	−1.48	4.00 × 10^−2^	−1.52	6.19 × 10^−3^	MYO16
chr14.0021177142	−1.22	3.13 × 10^−4^	−1.24	7.38 × 10^−5^	
chr14.0021316565	−1.29	2.23 × 10^−2^	−1.56	2.05 × 10^−4^	
chr14.0025947530	0.91	1.48 × 10^−2^	1.05	7.13 × 10^−3^	
chr14.0080449863	−1.84	5.11 × 10^−5^	−1.74	2.05 × 10^−4^	
chr14.0085404000	−1.38	4.50 × 10^−3^	−1.38	3.07 × 10^−3^	
chr14.0104190006	−1.48	3.71 × 10^−3^	−1.75	2.66 × 10^−4^	ZFYVE21
chr15.0046178808	−0.97	1.71 × 10^−2^	−0.70	4.78 × 10^−2^	RP11—718O11.1
chr15.0069824154	1.42	1.25 × 10^−4^	1.55	4.82 × 10^−6^	RP11—279F6.1
chr15.0092982723	1.45	3.91 × 10^−5^	1.73	9.39 × 10^−9^	ST8SIA2
chr16.0004815786	−0.89	8.94 × 10^−4^	−0.92	9.24 × 10^−3^	ZNF500
chr16.0077912976	−1.23	8.49 × 10^−3^	−1.28	2.22 × 10^−3^	VAT1L
chr16.0079468883	1.11	4.82 × 10^−3^	1.28	3.78 × 10^−5^	
chr17.0018941025	−1.85	1.91 × 10^−3^	−1.96	2.09 × 10^−4^	GRAP
chr17.0019045779	−1.55	7.74 × 10^−3^	−1.56	8.05 × 10^−4^	GRAPL, CTC—457L16.2
chr17.0028803808	−1.20	1.47 × 10^−2^	−1.24	4.72 × 10^−3^	
chr17.0070499160	1.04	1.42 × 10^−4^	1.06	1.48 × 10^−3^	LINC00511
chr17.0074566299	0.87	3.28 × 10^−2^	1.14	3.76 × 10^−4^	ST6GALNAC2, RP11—666A8.9
chr18.0043923940	−1.66	5.63 × 10^−6^	−1.73	7.53 × 10^−7^	RNF165
chr18.0045011716	1.10	2.73 × 10^−2^	1.41	4.57 × 10^−5^	CTD—2130O13.1
chr18.0047177650	−1.15	3.29 × 10^−4^	−0.64	8.52 × 10^−3^	
chr18.0047230566	−1.39	1.98 × 10^−2^	−1.30	3.48 × 10^−3^	
chr18.0072250823	1.12	7.11 × 10^−3^	1.05	2.58 × 10^−2^	CNDP1
chr19.0002867898	1.36	5.19 × 10^−9^	1.12	1.72 × 10^−2^	ZNF556
chr19.0041126191	−0.80	1.99 × 10^−2^	−0.92	4.84 × 10^−3^	LTBP4
chr20.0031210733	1.20	2.29 × 10^−2^	1.36	2.68 × 10^−2^	
chr20.0052825772	−1.35	1.91 × 10^−3^	−1.31	3.11 × 10^−3^	PFDN4
chr20.0055369320	−1.24	9.46 × 10^−4^	−1.68	1.37 × 10^−4^	
chr20.0060501154	2.02	6.08 × 10^−4^	1.87	9.34 × 10^−4^	CDH4
chr21.0030689317	−0.78	4.49 × 10^−3^	−0.86	1.16 × 10^−3^	BACH1
chr22.0050332646	−1.23	5.83 × 10^−3^	−1.35	1.32 × 10^−3^	

* LogFC = log_2_ fold change; positive logFC = hypermethylated in CP; negative logFC = hypomethylated in CP.

**Table 3:** Chromosome enrichment analysis *.

Chromosome	MB		MT		Muscle		Blood	
	Significant CpGs	Enrichment *p*-Value	Significant CpGs	Enrichment *p*-Value	Significant CpGs	Enrichment *p*-Value	Significant CpGs	Enrichment *p*-Value
1	0	1.000	1	1.000	1	1.000	10	1.000
2	26	0.997	103	1.000	84	0.361	312	1.000
3	45	4.81 ×10^−3^	112	0.146	77	9.94 × 10^−3^	650	2.20 × 10^−16^
4	3	1.000	9	1.000	7	1.000	21	1.000
5	14	0.998	82	0.863	64	0.082	222	1.000
6	29	0.348	98	0.209	109	1.22 × 10^−12^	544	2.20 × 10^−16^
7	8	1.000	23	1.000	23	1.000	136	1.000
8	9	1.000	26	1.000	28	0.999	169	1.000
9	18	0.921	54	1.000	22	1.000	208	1.000
10	13	0.999	35	1.000	4	1.000	76	1.000
11	33	0.103	101	0.092	58	0.196	618	2.20 × 10^−16^
12	76	2.20 × 10^−16^	204	2.20 × 10^−16^	67	7.43 × 10^−3^	519	2.20 × 10^−16^
13	21	3.73 × 10^−2^	104	7.37 × 10^−14^	61	9.60 × 10^−9^	344	2.20 × 10^−16^
14	41	3.31 × 10^−7^	137	2.20 × 10^−16^	68	6.01 × 10^−8^	448	2.20 × 10^−16^
15	31	1.63 × 10^−3^	130	2.20 × 10^−16^	72	6.96 × 10^−9^	298	3.44 × 10^−9^
16	20	0.835	93	0.111	68	3.04 × 10^−3^	369	1.37 × 10^−5^
17	26	0.564	86	0.659	56	0.329	300	0.912
18	35	4.65 × 10^−8^	107	2.20 × 10^−16^	47	1.42 × 10^−5^	341	2.20 × 10^−16^
19	9	1.000	47	1.000	25	1.000	170	1.000
20	47	4.59 × 10^−11^	153	2.20 × 10^−16^	64	4.93 × 10^−8^	484	2.20 × 10^−16^
21	2	0.995	6	1.000	2	1.000	9	1.000
22	19	0.101	63	1.09 × 10^−2^	31	0.257	293	2.20 × 10^−16^
Total	525		1774		1038		6541	

* Fisher’s exact test was used to determine chromosomes that contained an enrichment of differentially methylated CpGs. Significant *p*-values (*p* < 0.05) are indicated in **green**. Both cell conditions contained an enrichment of differentially methylated CpGs on the chromosomes indicated in **orange**. Significant CpG sites were enriched on the same chromosomes when analyzing muscle tissue and blood cells from subjects with CP.

**Table 4:** Significant promoter regions (+/− 1000 bp from TSS) in MBs *.

TSS	LogFC	FDR Corrected *p*-Value	Gene	Class
chr16:51277965	–0.85	3.82 × 10^−4^	AC137527.2	Pseudogene
chr13:115039303	0.20	1.97 × 10^−3^	MIR4502	miRNA
chr17:34397734	0.39	4.41 × 10^−2^	CCL18	Protein coding

* TSS = transcription start site, logFC = log_2_ fold change; positive logFC = hypermethylated in CP; negative logFC = hypomethylated in CP.

**Table 5:** Significant promoter regions (+/− 1000 bp from TSS) in MTs *.

TSS	LogFC	FDR Corrected *p*-Value	Gene	Class
chr17:73070401	0.59	9.50 × 10^−6^	AC111186.1	Pseudogene
chr17:75148756	0.36	4.01 × 10^−4^	RNU4–47P	snRNA
chr19:48673949	0.60	8.84 × 10^−4^	ZSWIM9	Protein coding
chr11:46134769	0.55	1.51 × 10^−3^	AC024475.1	miRNA
chr4:111866955	0.30	1.81 × 10^−3^	LYPLA1P2	Pseudogene
chr12:7072409	0.25	6.45 × 10^−3^	U47924.27	lincRNA
chr1:242187356	–0.14	6.93 × 10^−3^	RNU6–1139P	snRNA
chr12:7072408	0.25	7.37 × 10^−3^	EMG1	Protein coding
chr11:93971316	1.04	1.67 × 10^−2^	RP11–680H20.2	lincRNA
chr2:47454056	–0.67	4.96 × 10^−2^	AC106869.2	lincRNA

* TSS = transcription start site, logFC = log_2_ fold change; positive logFC = hypermethylated in CP; negative logFC = hypomethylated in CP.

## Data Availability

Methylomic data will be made publicly accessible via the controlled access system provided by the NIH database of Genotypes and Phenotypes (dbGaP) or can be made available upon a direct request to the corresponding author.

## Supplementary Materials

The following supporting information can be downloaded at https://www.mdpi.com/article/10.3390/jpm12121978/s1, Figure S1: Cell morphology, Figure S2: Scree plots for explained variance, Figure S3: Volcano plots, Figure S4: Manhattan plots, Table S1: Cell culture information, Table S2: Significant CpGs in MBs, Table S3: Significant CpGs in MTs.
