# Supplementary material for: DNA Methylation Analysis Reveals Distinct Patterns in Satellite Cell–Derived Myogenic Progenitor Cells of Subjects with Spastic Cerebral Palsy
Source: J Pers Med. 2022 Nov 30;12(12):1978. doi: 10.3390/jpm12121978 (PMC9780849; doi:10.3390/jpm12121978)
Supplement: Supplementary file 1 [file jpm-12-01978-s001.zip › jpm-2073048-supplementary.pdf]

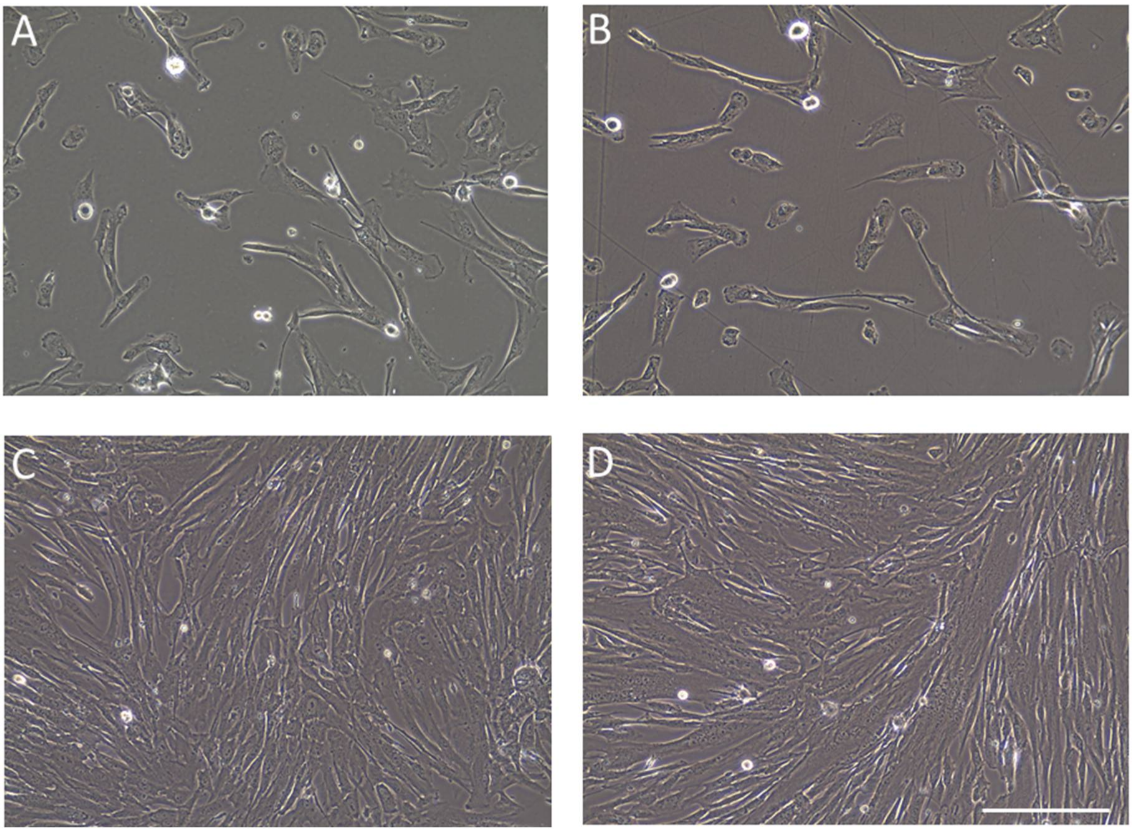

Figure S1: Cell morphology. Skeletal muscle biopsies underwent enzymatic digestion and subsequent double-immunomagnetic isolation to collect a population of SCs. Proliferating MBs were collected at 50% confluence. Cultures were differentiated in low-serum medium for 24 hours to initiate myoblast fusion (MTs). Representative images of control MBs after 72 hours of proliferation **(A)**, CP MBs after 72 hours of proliferation **(B)**, control MTs after 24 hours of differentiation **(C)**, and CP MTs after 24 hours of differentiation **(D)** are shown. Cells derived from subjects with CP had similar morphology to those derived from controls. Scale bar = 200 μm.

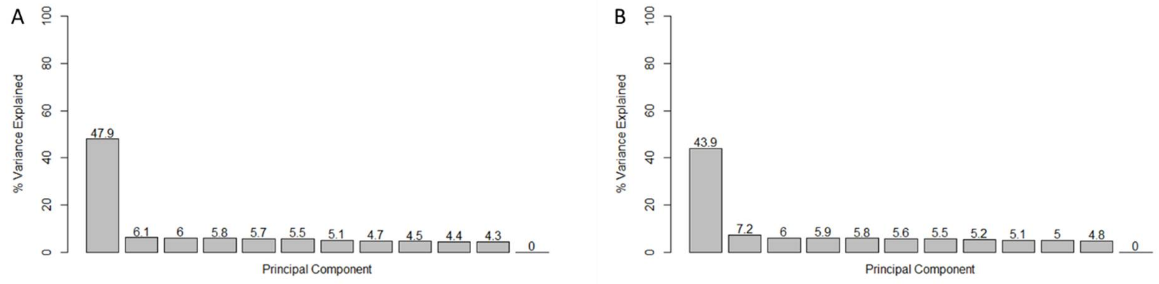

Figure S2: Scree plots for explained variance. Plots were generated to depict how much variation each principal component captured from the data for MBs **(A)** and MTs **(B)**. The number of components are plotted on the x-axis against the percent of the variance explained on the y-axis. For both MBs and MTs, the first two principal components described more than 50% of the variance and were used for PCA.

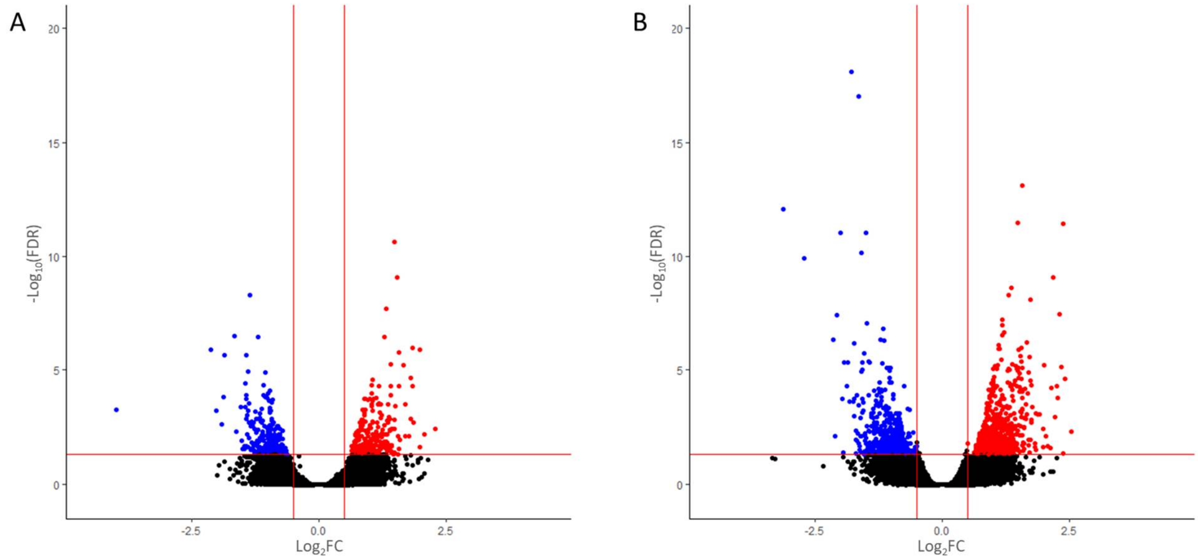

Figure S3: Volcano plots. The frequency profile of p-values is shown for MBs **(A)** and MTs **(B)**. Data from the ~1.48 million CpG sites in common across all samples are plotted. The x-axis is the $\log_2$ value for the fold change (ratio) of CP to non-CP CpG site methylation values and the y-axis is the negative $\log_{10}$ FDR value. Red lines indicate LogFC greater than 0.5 or less than -0.5 and FDR < 0.05 (black = not significant, red = significantly hypermethylated in CP, blue = significantly hypomethylated in CP).

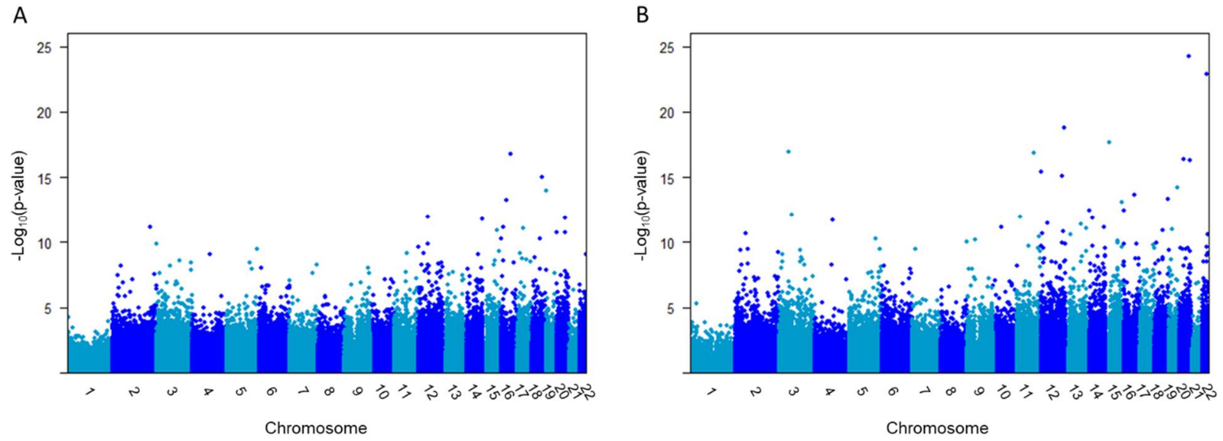

Figure S4: Manhattan plots. The negative logarithm of the p-value versus the chromosomal location for each of the ~1.48 million CpG sites is shown for MBs **(A)** and MTs **(B)**.

Table S1. Cell culture information for each sample.

Sample	PAX7+ %	Passage Number	Days to MB Collection
CN1	98	3	5
CN2	97	4	2
CN3	99	4	2
CN5	99	4	3
CN4	98	4	3
CN6	98	5	3
**CN Mean**	**98.2**	**4**	**3.0**
CP4	92	3	3
CP3	100	3	2
CP2	100	5	3
CP5	97	3	3
CP6	99	4	3
CP1	90	3	4
**CP Mean**	**96.3**	**3.5**	**3.0**

Table S2. Significant CpGs in MBs. LogFC = log_2 fold change; positive logFC = hypermethylated in CP; negative logFC = hypomethylated in CP; blank values for gene = intergenic regions. X's indicate sites within muscle genes or annotated enhancers.

CpG site	LogFC	FDR corrected p-value	Gene	Muscle Gene	Enhancer Region
chr16.0051277978	-1.48	2.30E-11	AC137527.2		
chr18.0055106886	-1.54	7.91E-10	ONECUT2		
chr19.0002867898	1.36	5.17E-09	ZNF556		
chr16.0029232332	-1.33	2.05E-08			
chr14.0104759275	1.66	3.34E-07			
chr12.0053812491	-1.29	3.37E-07			
chr20.0045674015	1.19	3.47E-07	EYA2		
chr16.0014797680	-1.83	1.09E-06	RP11-82O18.2		
chr2.0207023289	-1.97	1.22E-06	NDUFS1		
chr17.0034503322	2.13	1.24E-06	CTB-91J4.1, TBC1D3B		
chr15.0078609028	-1.58	1.66E-06			
chr20.0046478296	1.42	2.19E-06			
chr20.0000837287	1.85	2.25E-06	FAM110A		
chr16.0003062975	-1.42	5.61E-06	CLDN9		
chr18.0043923940	-1.66	5.84E-06	RNF165		
chr3.0001897820	1.39	1.20E-05			
chr12.0049484968	1.05	1.25E-05	DHH, RP11-386G11.8		
chr12.0001356415	-1.80	2.10E-05	ERC1		
chr5.0171527362	-1.05	2.61E-05	STK10		
chr15.0092982723	1.45	3.90E-05	ST8SIA2		
chr11.0070369682	1.09	4.71E-05	SHANK2		
chr4.0101719592	-1.04	4.72E-05	EMCN		
chr22.0051206404	-1.17	4.91E-05	RPL23AP82, RABL2B		
chr17.0014203257	-1.57	5.05E-05			
chr12.0025342500	-1.40	5.06E-05	CASC1		X
chr14.0080449863	-1.84	5.09E-05			X
chr18.0012775069	0.97	7.79E-05	RP11-973H7.1		
chr17.0029150027	-1.04	1.02E-04	CRLF3		
chr17.0049009115	1.00	1.08E-04			
chr16.0013247532	1.08	1.25E-04	SHISA9		X
chr3.0127606140	-1.40	1.26E-04			
chr15.0069824154	1.42	1.26E-04	RP11-279F6.1		
chr17.0070499160	1.04	1.42E-04	LINC00511		
chr5.0133179996	1.06	1.53E-04			X
chr16.0067562199	1.87	1.54E-04	FAM65A		
chr12.0124320029	1.01	1.58E-04	DNAH10		

chr3.0195923929	-1.13	1.58E-04			
chr12.0114887843	1.42	1.83E-04			
chr12.0016716975	-0.90	1.85E-04	MGST1, LMO3		
chr15.0059214432	-1.06	1.86E-04	RNF111, SLTM		
chr12.0109189987	-1.00	1.88E-04	SSH1		
chr12.0051864464	-0.90	1.89E-04	SLC4A8		
chr7.0149155842	0.90	2.00E-04	ZNF777		
chr12.0054428934	-0.97	2.05E-04	HOXC4, RP11-834C11.14, HOXC5		
chr2.0046167919	-1.06	2.14E-04	PRKCE		
chr3.0075657744	0.97	2.18E-04			
chr12.0131408548	0.92	2.57E-04			
chr6.0016145414	-1.17	2.93E-04	MYLIP		
chr9.0133233691	1.36	2.96E-04	HMCN2		
chr14.0100204041	-1.35	3.04E-04	EML1		
chr14.0021177142	-1.22	3.12E-04			
chr12.0048360477	-1.69	3.15E-04	TMEM106C		
chr5.0141817569	-1.04	3.21E-04	AC005592.2		
chr18.0047177650	-1.15	3.28E-04			X
chr18.0077704208	-1.50	3.65E-04	PQLC1		
chr3.0195630444	-1.04	3.92E-04	TNK2		
chr12.0032051620	1.52	4.01E-04			
chr20.0048644582	1.44	4.25E-04			
chr11.0065923292	0.97	4.58E-04	PACS1		
chr12.0119746133	0.97	4.62E-04			
chr13.0060208310	1.12	4.92E-04			
chr3.0017817015	-0.90	5.51E-04	TBC1D5		
chr19.0048414587	3.97	5.53E-04			
chr7.0129412947	-0.85	5.66E-04			
chr12.0101056159	0.97	5.72E-04			
chr9.0139029439	1.38	5.78E-04			X
chr12.0056332772	0.96	5.87E-04	DGKA		
chr13.0036266089	0.94	6.00E-04			
chr20.0060501154	2.02	6.06E-04	CDH4		
chr2.0235215325	-1.05	6.11E-04			
chr2.0029850455	1.24	6.71E-04	ALK		
chr13.0108997650	-1.20	7.03E-04			
chr20.0024608543	0.95	7.34E-04	SYNDIG1		
chr12.0127541139	-0.89	7.96E-04	RP11-575F12.1		
chr14.0035591938	1.15	8.20E-04	KIAA0391		
chr16.0004815786	-0.89	8.91E-04	ZNF500		
chr20.0062778083	1.41	9.33E-04			

chr12.0120338446	-0.90	9.43E-04			
chr20.0055369320	-1.24	9.54E-04			
chr12.0121475432	-0.90	9.70E-04	OASL		
chr11.0123045794	-1.36	1.12E-03	CLMP		
chr2.0110054189	1.16	1.24E-03	SH3RF3		
chr10.0081716405	-1.20	1.30E-03	SFTPD		
chr14.0069029827	1.42	1.36E-03	RAD51B		
chr13.0112756716	-1.40	1.37E-03			
chr14.0105855078	1.24	1.37E-03	PACS2		
chr10.0116680012	0.92	1.38E-03			
chr14.0073925622	-1.79	1.40E-03	NUMB		
chr18.0021282448	-0.84	1.46E-03	LAMA3		
chr14.0096510341	-0.86	1.47E-03	C14orf132		
chr7.0002555562	1.16	1.51E-03	LFNG		
chr13.0107190658	-1.48	1.52E-03			
chr3.0112241221	-0.87	1.62E-03			
chr15.0069678825	1.10	1.66E-03	PAQR5		
chr12.0056048792	0.93	1.67E-03	RP11-644F5.16, RP11-644F5.15		
chr18.0011021280	-1.34	1.72E-03	PIEZO2		X
chr11.0062248057	1.14	1.77E-03	AHNAK		
chr2.0056193463	1.32	1.78E-03	RP11-481J13.1, AC011306.2		
chr9.0092292403	-1.06	1.85E-03			
chr14.0094883780	-0.90	1.86E-03			
chr3.0060919598	-0.85	1.88E-03	FHIT		
chr10.0130653464	1.36	1.91E-03			
chr12.0095901262	1.12	1.91E-03	METAP2		
chr17.0018941025	-1.85	1.92E-03	GRAP		
chr14.0080475557	0.89	1.92E-03			
chr20.0052825772	-1.35	1.93E-03	PFDN4		
chr20.0003110156	-0.80	1.94E-03	UBOX5-AS1, UBOX5		
chr14.0038206052	-1.05	1.98E-03	TTC6		
chr3.0119863345	1.28	2.04E-03	GPR156		
chr3.0119990864	-1.14	2.04E-03	GPR156		
chr13.0027580970	1.91	2.33E-03			
chr3.0107294807	-0.83	2.35E-03	BBX		
chr15.0079214816	0.93	2.35E-03	CTSH		
chr22.0019752899	0.91	2.35E-03	TBX1	X	
chr9.0036492536	-0.86	2.53E-03			
chr12.0072524007	0.97	2.62E-03	TPH2, TRHDE		
chr18.0011467550	-0.99	2.63E-03	RP11-128P17.3		
chr6.0043541893	-1.28	2.70E-03	XPO5		

chr6.0052623375	1.33	2.91E-03	GSTA2		
chr6.0170399029	0.82	2.92E-03			
chr14.0076248154	-0.81	2.93E-03	TTLL5		
chr2.0241908694	1.07	2.94E-03	AC104809.3		
chr6.0019413218	0.81	2.94E-03			
chr20.0056593028	1.31	2.95E-03			
chr18.0033355015	0.99	3.03E-03			
chr20.0062083383	-1.16	3.19E-03	KCNQ2		
chr17.0038462448	1.03	3.23E-03			
chr2.0033057636	-0.84	3.39E-03	LINC00486		
chr12.0002910617	-1.01	3.39E-03	FKBP4, RP4-816N1.6, RP4-816N1.7		
chr11.0127179783	0.84	3.50E-03	RP11-480C22.1		
chr20.0061968208	1.26	3.62E-03			
chr14.0104190006	-1.48	3.70E-03	ZFYVE21		
chr6.0161063597	-2.29	3.71E-03	LPA		
chr6.0168745680	0.92	3.72E-03			
chr10.0114569149	0.77	3.72E-03	VTI1A		
chr9.0139231307	-0.83	3.73E-03	GPSM1		
chr12.0018457486	-1.42	3.75E-03	RERGL, PIK3C2G		
chr12.0105147522	-0.97	3.84E-03	CHST11		
chr18.0030805847	-0.82	4.17E-03	CCDC178		
chr15.0101632053	-1.25	4.17E-03			
chr14.0104733862	-0.84	4.25E-03	RP11-260M19.2		
chr3.0141524375	-1.39	4.26E-03	GRK7		
chr12.0003241735	1.19	4.26E-03	TSPAN9		
chr12.0110324807	-0.79	4.28E-03	RP1-7G5.6		
chr14.0083607338	0.97	4.29E-03			
chr2.0242587453	0.86	4.31E-03	ATG4B		
chr12.0016028478	-0.94	4.46E-03	EPS8		
chr11.0120302298	-0.76	4.47E-03	ARHGEF12		
chr21.0030689317	-0.78	4.48E-03	BACH1		
chr14.0085404000	-1.38	4.49E-03			
chr16.0028329253	-0.90	4.50E-03	SBK1		
chr11.0089386768	0.72	4.50E-03	FOLH1B		
chr6.0035412073	1.00	4.69E-03	MKRNP2		
chr16.0079468883	1.11	4.80E-03			
chr12.0083436417	1.62	4.93E-03	TMTC2		
chr14.0102685905	-1.13	4.96E-03	WDR20		
chr22.0018624236	-0.79	4.96E-03	TUBA8		
chr18.0021082035	1.00	5.00E-03			
chr11.0018392462	-0.72	5.23E-03			

chr3.0033354536	-0.97	5.42E-03	FBXL2		
chr20.0046839598	1.22	5.59E-03			
chr20.0003681200	0.95	5.65E-03	SIGLEC1		
chr12.0007270902	-0.94	5.78E-03	C1RL-AS1		
chr22.0050332646	-1.23	5.81E-03			
chr13.0033220266	-1.24	5.85E-03	PDS5B		
chr2.0101235133	-0.77	5.88E-03			
chr12.0003412978	0.77	6.31E-03			
chr18.0019179732	-2.07	6.32E-03	ESCO1		
chr22.0043245863	-0.73	6.43E-03	ARFGAP3, PACSIN2		
chr14.0023826830	-1.12	6.45E-03	EFS		
chr11.0108812687	-0.71	6.48E-03			
chr16.0067003618	0.78	6.49E-03	CES3		
chr14.0070145650	-1.06	6.50E-03	KIAA0247		
chr13.0047191668	-1.30	6.51E-03	LRCH1		
chr20.0061078379	1.11	6.52E-03			
chr17.0017713313	0.96	6.52E-03	RAI1		
chr12.0002464403	-1.33	6.55E-03	CACNA1C	X	
chr16.0020279909	-1.11	6.55E-03			X
chr18.0045207364	1.40	6.71E-03			
chr12.0064441275	-1.21	6.72E-03	SRGAP1, RP11-196H14.2		
chr9.0124320846	0.87	6.75E-03			
chr6.0036400034	1.02	7.00E-03	PXT1		
chr18.0072250823	1.12	7.08E-03	CNDP1		
chr3.0013864864	-0.72	7.10E-03	WNT7A		
chr22.0047857822	-0.96	7.57E-03	LL22NC03-75H12.2		
chr12.0116630659	-0.79	7.58E-03	MED13L		
chr9.0115938896	0.79	7.60E-03	FKBP15		X
chr14.0056763462	-1.14	7.60E-03	PELI2		
chr16.0074454096	0.91	7.61E-03	CLEC18B		
chr17.0019045779	-1.55	7.71E-03	GRAPL, CTC-457L16.2		
chr13.0107176083	-1.69	7.73E-03	EFNB2		
chr3.0046499569	-0.84	7.84E-03	LTF		
chr11.0009112240	-0.79	7.88E-03	SCUBE2		
chr19.0045378240	0.87	8.05E-03	PVRL2		
chr11.0062533046	-0.77	8.09E-03	POLR2G		
chr3.0046979919	1.08	8.14E-03	CCDC12		
chr12.0069834797	0.98	8.21E-03			
chr15.0037402654	-1.76	8.22E-03			
chr2.0028466430	-0.76	8.33E-03	BRE		
chr3.0197251389	-0.77	8.35E-03	BDH1		

chr16.0077912976	-1.23	8.46E-03	VAT1L		
chr18.0056701704	0.98	8.79E-03	OACYLP		
chr10.0103757273	0.77	8.81E-03	C10orf76		
chr22.0050305243	-0.78	8.83E-03	ALG12		
chr11.0008743403	1.15	9.04E-03	ST5		
chr10.0020132012	0.77	9.14E-03	PLXDC2		
chr8.0041168893	-1.07	9.31E-03			
chr4.0166097490	-0.74	9.33E-03	TMEM192		
chr6.0154640863	1.37	9.64E-03	IPCEF1		
chr22.0050620859	-0.77	9.89E-03			
chr20.0062049798	-1.08	9.89E-03			
chr2.0071864693	-1.13	9.98E-03	DYSF	X	
chr18.0019601842	0.99	1.00E-02	RP11-595B24.1		
chr18.0020836978	0.98	1.00E-02	CABLES1, TMEM241		
chr12.0039914797	-0.89	1.00E-02			
chr2.0035092870	-1.09	1.00E-02	AC012593.1		
chr5.0115935698	0.74	1.01E-02			
chr15.0024672858	1.12	1.02E-02			
chr17.0036416584	0.73	1.03E-02	RP11-1407O15.2		
chr5.0166472226	-1.03	1.03E-02			
chr3.0038031943	-0.79	1.03E-02	VILL		
chr3.0126094904	0.79	1.06E-02			X
chr12.0129876695	0.73	1.08E-02	TMEM132D		
chr3.0046643520	0.71	1.08E-02	TDGF1		
chr3.0062838161	0.81	1.09E-02	CADPS		
chr8.0038241237	-0.77	1.09E-02			
chr20.0025442435	0.91	1.13E-02	NINL		
chr13.0071457891	0.84	1.14E-02			
chr20.0033883939	-1.58	1.17E-02			
chr6.0122837153	-0.78	1.17E-02	PKIB		
chr14.0023701059	0.79	1.17E-02	C14orf164		
chr17.0002235852	-0.73	1.17E-02	TSR1		
chr7.0016768868	-0.75	1.18E-02			
chr8.0041168336	1.09	1.18E-02			
chr20.0059528314	-1.06	1.18E-02			
chr20.0043335999	1.21	1.20E-02	RP11-445H22.4		
chr15.0061267563	0.98	1.20E-02	RORA		
chr14.0028616430	0.90	1.20E-02			
chr22.0045949021	-1.04	1.20E-02	FBLN1		
chr16.0084205031	-0.93	1.21E-02	DNAAF1		
chr9.0038111399	0.88	1.21E-02			

chr18.0007824130	-1.16	1.21E-02	PTPRM		
chr11.0067273409	1.09	1.22E-02	PITPNM1		
chr13.0027424109	1.51	1.22E-02			X
chr6.0007447123	-1.02	1.29E-02			
chr15.0038006605	0.89	1.30E-02			
chr12.0027821943	-0.99	1.30E-02	PPFIBP1		
chr20.0003441625	-1.28	1.31E-02			
chr19.0004634744	0.98	1.32E-02			
chr12.0039929800	-1.20	1.35E-02			
chr20.0037481710	-0.90	1.37E-02	PPP1R16B		
chr11.0134296018	1.17	1.37E-02			
chr9.0101561563	-0.72	1.38E-02			
chr12.0111017742	-0.70	1.38E-02	PPTC7		
chr14.0067989640	-1.12	1.39E-02	TMEM229B		
chr16.0011016948	-0.96	1.40E-02	CIITA		
chr12.0118509696	-0.73	1.41E-02	VSIG10		
chr12.0116068191	-1.32	1.41E-02	RP11-1028N23.4		
chr17.0028803808	-1.20	1.47E-02			
chr14.0025947530	0.91	1.48E-02			
chr22.0020613685	0.86	1.48E-02	AC011718.1		
chr12.0009243798	0.88	1.48E-02	A2M		
chr11.0047489314	0.98	1.49E-02	CELF1		
chr11.0090180873	1.32	1.49E-02	DISC1FP1		
chr12.0109848872	-0.97	1.51E-02	MYO1H		
chr3.0189791239	-1.34	1.55E-02	LEPREL1		
chr18.0075332957	1.22	1.55E-02			
chr15.0021241379	0.84	1.56E-02			
chr3.0053784559	0.91	1.57E-02	CACNA1D	X	
chr14.0061385863	-1.02	1.58E-02	MNAT1, RP11-193F5.1		
chr11.0129565594	1.28	1.59E-02			
chr11.0016936211	0.88	1.59E-02	PLEKHA7		
chr20.0043953416	1.02	1.59E-02			
chr7.0044621160	0.91	1.71E-02	TMED4		
chr3.0176960084	-0.69	1.71E-02			
chr15.0046178808	-0.97	1.71E-02	RP11-718O11.1		
chr11.0131850680	0.88	1.74E-02	NTM		
chr3.0194597824	-1.29	1.74E-02			
chr9.0123508761	-0.85	1.74E-02			
chr20.0001916618	-1.25	1.78E-02	SIRPA		
chr9.0019169503	0.73	1.79E-02			
chr14.0075694331	-0.89	1.83E-02			

chr18.0033652604	0.97	1.84E-02			
chr11.0127193165	0.72	1.90E-02			
chr13.0024636355	-1.10	1.93E-02	RP11-307N16.6, SPATA13		
chr12.0123727933	-1.03	1.93E-02	MPHOSPH9, C12orf65		
chr20.0004374415	-1.43	1.94E-02			
chr18.0047230566	-1.39	1.98E-02			X
chr3.0013175389	-1.04	1.99E-02			
chr19.0041126191	-0.80	1.99E-02	LTBP4		
chr18.0053360011	1.01	2.00E-02			
chr5.0155939097	-0.86	2.03E-02	SGCD	X	
chr11.0046403972	1.06	2.03E-02	MDK		
chr6.0167402538	-0.72	2.07E-02	RP1-167A14.2		
chr16.0069786693	0.75	2.07E-02	NOB1		
chr12.0056095087	-1.17	2.13E-02	ITGA7	X	
chr12.0114047563	-1.42	2.18E-02			
chr12.0131689822	1.29	2.18E-02	RP11-638F5.1		
chr3.0124562223	0.98	2.19E-02	ITGB5	X	
chr15.0090525709	1.46	2.19E-02			
chr22.0050349213	1.20	2.19E-02			
chr3.0009739416	-0.89	2.20E-02	MTMR14		
chr20.0039452845	-0.95	2.21E-02			
chr3.0197237955	-0.81	2.21E-02			
chr14.0021316565	-1.29	2.22E-02			
chr20.0059724480	1.42	2.22E-02			
chr17.0072850038	0.81	2.22E-02	GRIN2C		
chr11.0131635593	-0.82	2.22E-02			
chr22.0023191105	0.74	2.22E-02			
chr11.0070808370	-0.64	2.24E-02			
chr14.0023876911	-1.08	2.25E-02	MYH6	X	
chr12.0008027124	0.85	2.26E-02	SLC2A14		
chr13.0020794750	0.98	2.26E-02			
chr6.0008948266	1.16	2.27E-02			
chr9.0131024026	-0.69	2.27E-02	GOLGA2		
chr12.0117047937	-0.69	2.28E-02			
chr18.0021435392	0.93	2.28E-02			
chr6.0031008851	0.96	2.29E-02	RASSF3		
chr12.0065005764	-1.34	2.29E-02			
chr11.0000821322	1.38	2.29E-02	PNPLA2		
chr3.0034906342	-0.85	2.29E-02			X
chr5.0100843674	0.73	2.29E-02			
chr7.0016504755	0.80	2.29E-02	SOSTDC1		

chr19.0049703565	-0.73	2.29E-02	TRPM4		
chr14.0104119867	-1.98	2.29E-02	KLC1, RP11-73M18.2		
chr20.0031210733	1.20	2.29E-02			
chr2.0003882321	0.88	2.29E-02			
chr15.0055228849	1.03	2.29E-02			
chr20.0013130466	1.04	2.30E-02	SPTLC3		
chr6.0010385427	-1.35	2.31E-02			
chr3.0134320672	-0.92	2.37E-02	EPHB1		
chr13.0044928237	-0.87	2.39E-02			
chr3.0148627752	0.89	2.40E-02	RP11-680B3.2		
chr10.0003512410	-0.97	2.43E-02			
chr3.0103216920	0.78	2.46E-02			
chr20.0031015233	-0.83	2.47E-02	ASXL1		
chr5.0011534641	0.90	2.48E-02	CTNND2		
chr20.0020806502	-0.90	2.48E-02			
chr9.0137156003	0.90	2.48E-02			
chr18.0057103282	0.93	2.48E-02	CCBE1		
chr15.0069901941	0.91	2.48E-02	RP11-279F6.3		
chr14.0105384756	1.30	2.49E-02			
chr8.0042348640	-1.02	2.50E-02	SLC20A2		
chr10.0011491536	0.71	2.50E-02			
chr5.0164483805	-0.92	2.50E-02	CTC-340A15.2		
chr22.0025365026	0.83	2.52E-02	KIAA1671		
chr10.0024327589	-0.87	2.52E-02	KIAA1217		
chr12.0117143952	-0.82	2.53E-02			
chr14.0036939568	1.00	2.58E-02			
chr20.0049033760	-1.31	2.61E-02			
chr12.0085683529	0.95	2.62E-02	ALX1		
chr22.0029706927	-1.24	2.62E-02	GAS2L1		
chr20.0062149183	-1.00	2.62E-02			
chr12.0130994695	-0.95	2.63E-02	RIMBP2		
chr17.0070895822	-0.68	2.66E-02	SLC39A11		
chr11.0066622735	0.65	2.66E-02	PC		
chr6.0002286209	0.95	2.70E-02	GMDS-AS1		
chr15.0027477685	-1.00	2.71E-02	GABRG3		
chr3.0048310078	1.02	2.71E-02	ZNF589		
chr13.0023270862	0.77	2.71E-02			
chr18.0045011716	1.10	2.72E-02	CTD-2130O13.1		
chr18.0059531531	-0.78	2.72E-02	RNF152		
chr9.0109806856	-0.92	2.73E-02	RP11-508N12.2		
chr8.0058130120	-0.70	2.73E-02	RP11-513O17.2		

chr12.0124936839	-1.05	2.76E-02	NCOR2		
chr16.0079745969	0.88	2.79E-02	RP11-345M22.1		
chr3.0033102830	-0.84	2.79E-02	GLB1		
chr17.0010478124	0.73	2.80E-02	CTC-297N7.11		
chr13.0033684630	1.46	2.84E-02	STARD13		
chr10.0015273527	-0.70	2.85E-02	FAM171A1		
chr15.0097407473	-0.82	2.89E-02			
chr20.0060186448	-1.56	2.93E-02			
chr17.0061612901	0.79	2.93E-02	KCNH6		
chr15.0053264991	-0.91	2.93E-02			
chr2.0242102221	-0.94	2.94E-02	PPP1R7		
chr13.0093896533	1.50	2.95E-02	GPC6		
chr18.0070751712	-0.97	2.96E-02			
chr3.0158464604	0.67	2.99E-02	MFSD1, RP11-379F4.4		
chr15.0045111673	-1.20	3.00E-02			
chr15.0090574827	1.15	3.02E-02	ZNF710		
chr5.0072485778	1.10	3.02E-02			
chr15.0057858330	0.90	3.03E-02			
chr17.0077808736	0.93	3.03E-02	CBX4		
chr12.0096032685	1.34	3.04E-02			
chr2.0230915720	0.85	3.04E-02	SLC16A14		
chr12.0094153261	1.13	3.06E-02	CRADD		
chr12.0110028502	-0.87	3.06E-02	MVK		
chr22.0049023822	0.74	3.10E-02	FAM19A5		X
chr9.0095057658	-0.90	3.10E-02			
chr8.0027800853	0.67	3.11E-02	SCARA5		
chr14.0073426628	-0.98	3.18E-02			
chr6.0123159641	0.88	3.20E-02			
chr12.0117440300	-0.90	3.21E-02	FBXW8		
chr2.0241639679	1.27	3.23E-02			
chr12.0128167651	1.13	3.27E-02			
chr11.0065933711	0.79	3.27E-02			
chr19.0042929177	0.70	3.27E-02	LIPE-AS1, LIPE, CTB-50E14.4		
chr20.0042729620	-1.11	3.27E-02			
chr15.0068286485	-0.85	3.28E-02			
chr17.0074566299	0.87	3.28E-02	ST6GALNAC2, RP11-666A8.9		
chr16.0010390919	0.91	3.28E-02			
chr12.0056333039	0.90	3.28E-02			
chr7.0147581299	-0.68	3.29E-02	CNTNAP2		
chr6.0025139941	-1.02	3.32E-02	CMAHP		
chr12.0050500877	0.83	3.32E-02	GPD1		

chr15.0093801101	0.74	3.32E-02	RP11-326A13.1		
chr6.0037514594	0.84	3.32E-02	RP1-153P14.5, RP1-153P14.3		
chr18.0048375821	-1.04	3.33E-02			
chr3.0055881684	-0.88	3.33E-02	ERC2		
chr9.0068235180	0.63	3.33E-02			
chr14.0075405758	-0.91	3.37E-02			
chr12.0056755177	-1.17	3.38E-02	APOF		
chr14.0029181235	0.79	3.39E-02			
chr20.0052351842	-1.01	3.42E-02			
chr22.0037334249	-0.76	3.47E-02	CSF2RB		
chr5.0116309052	0.81	3.50E-02			
chr9.0121931878	-1.35	3.50E-02	BRINP1		
chr14.0060810689	0.98	3.51E-02			
chr17.0052639919	-1.12	3.51E-02			
chr11.0017832522	-0.95	3.51E-02	SERGEF		
chr20.0057144398	-1.05	3.52E-02	APCDD1L-AS1		
chr17.0037449771	-0.64	3.53E-02	FBXL20		
chr3.0196595774	-1.37	3.53E-02	SENP5		
chr13.0099687193	1.01	3.53E-02	DOCK9		
chr14.0102174978	-1.41	3.55E-02	RP11-1029J19.5		
chr3.0168473460	-0.88	3.55E-02	EGFEM1P		
chr18.0002417135	-0.87	3.55E-02			
chr18.0060803911	-1.13	3.55E-02	BCL2		
chr17.0035398896	-0.69	3.57E-02	AATF, CTC-268N12.3		
chr14.0101254911	-0.75	3.58E-02	MEG3		
chr4.0068334446	0.66	3.59E-02			X
chr8.0099957010	-1.09	3.64E-02	OSR2		
chr6.0083334853	0.66	3.65E-02			
chr15.0039742794	-0.76	3.65E-02			
chr6.0134589430	-1.04	3.73E-02	SGK1		
chr15.0093177616	1.01	3.75E-02	FAM174B		
chr12.0124895363	0.97	3.76E-02			
chr20.0062550242	-0.78	3.80E-02	DNAJC5		X
chr18.0022623623	-0.91	3.81E-02			
chr3.0182124231	-1.14	3.81E-02			X
chr20.0041428080	0.87	3.81E-02	PTPRT		
chr12.0120727765	-1.34	3.82E-02			
chr2.0148097446	0.70	3.83E-02			
chr17.0039250188	0.80	3.83E-02			
chr2.0009912902	-0.70	3.83E-02			
chr2.0217502199	0.87	3.87E-02	IGFBP2		

chr2.0232111330	0.69	3.87E-02	ARMC9		
chr5.0039219698	1.22	3.90E-02	FYB		
chr20.0013332818	0.88	3.90E-02	TASP1		
chr6.0148815687	0.99	3.92E-02	SASH1		
chr17.0015366928	1.10	3.92E-02	CDRT4, TVP23C-CDRT4, TVP23C		
chr5.0108133030	-0.71	3.96E-02	FER		
chr12.0085862864	-1.18	3.98E-02			
chr15.0069686871	1.17	3.98E-02			
chr17.0017096165	-1.00	3.99E-02	MPRIP		
chr13.0109856377	-1.48	3.99E-02	MYO16		X
chr22.0043582319	1.08	3.99E-02	TTLL12		
chr20.0030641297	0.99	4.00E-02	HCK		
chr3.0050412109	-0.83	4.01E-02	CACNA2D2		
chr14.0094511641	-0.90	4.03E-02	OTUB2		
chr16.0084618353	0.93	4.03E-02	COTL1		
chr9.0003201208	-0.79	4.07E-02			
chr2.0223166989	0.87	4.07E-02	CCDC140		
chr12.0104998453	1.26	4.21E-02			
chr20.0058031564	0.93	4.22E-02			
chr3.0112947928	-1.11	4.23E-02	BOC		
chr6.0037113416	-0.73	4.24E-02			
chr14.0073118040	-1.42	4.26E-02	DPF3		
chr11.0122086249	1.23	4.29E-02	RP11-820L6.1		
chr19.0013700380	1.10	4.31E-02	CACNA1A		
chr10.0125233252	0.82	4.34E-02	RP11-282I1.1		
chr12.0055783991	1.19	4.34E-02			
chr8.0142865287	0.64	4.34E-02			
chr2.0225889464	-1.00	4.35E-02	DOCK10		
chr20.0030177138	-0.93	4.35E-02			
chr22.0033790923	0.66	4.36E-02	LARGE1	X	
chr15.0051979269	0.92	4.43E-02	SCG3		
chr8.0145165331	-0.68	4.45E-02	KIAA1875		
chr11.0125226340	0.78	4.50E-02	PKNOX2		
chr19.0005142189	-0.80	4.50E-02	KDM4B		
chr17.0040253756	1.14	4.52E-02	DHX58		
chr13.0031926541	-1.14	4.53E-02			
chr7.0137873425	0.67	4.55E-02			
chr20.0009574232	-0.91	4.58E-02	PAK7		
chr11.0064061447	0.69	4.58E-02	KCNK4, RP11-783K16.10		
chr12.0057404060	0.83	4.58E-02	TAC3		
chr2.0016179024	0.94	4.59E-02	AC010145.4		

chr15.0057660329	0.97	4.62E-02			
chr15.0028821466	-1.10	4.62E-02	RP11-665A22.1		
chr6.0017105598	0.86	4.62E-02	STMND1		
chr10.0104366434	-0.69	4.62E-02	SUFU		
chr12.0022379973	-0.99	4.63E-02	ST8SIA1		
chr22.0039679677	0.76	4.63E-02			
chr6.0168744278	-0.73	4.65E-02			
chr20.0045382067	-0.86	4.65E-02			
chr12.0133655015	0.83	4.65E-02			
chr18.0045856283	-0.83	4.65E-02	ZBTB7C		
chr12.0026672531	1.00	4.65E-02	ITPR2		
chr12.0050232608	0.94	4.66E-02	BCDIN3D-AS1, BCDIN3D		
chr5.0071798672	-0.89	4.66E-02	ZNF366		
chr2.0238314153	0.90	4.69E-02	COL6A3	X	
chr6.0148174138	-0.88	4.76E-02	RP11-307P5.1		
chr16.0000331917	0.83	4.76E-02	ARHGDIG		
chr14.0070317146	-1.21	4.77E-02			
chr11.0067345684	-1.16	4.77E-02			
chr12.0063894317	1.02	4.80E-02			
chr21.0044609126	0.86	4.81E-02			
chr6.0147770007	0.62	4.82E-02			
chr6.0036011510	-0.89	4.82E-02	MAPK14	X	
chr20.0058218260	0.93	4.86E-02	PHACTR3		
chr10.0093474401	-0.65	4.86E-02			
chr11.0011584859	-0.85	4.86E-02	GALNT18		X
chr12.0049982264	-0.90	4.86E-02	PRPF40B, FAM186B		
chr13.0021286449	-1.10	4.86E-02	IL17D		
chr17.0059573051	0.97	4.86E-02			
chr11.0031811526	0.64	4.87E-02	PAX6		
chr3.0010978002	0.74	4.87E-02	SLC6A11		X
chr18.0013924980	-1.46	4.88E-02			
chr12.0054366343	0.87	4.92E-02	HOTAIR		
chr18.0043684277	0.99	4.94E-02	ATP5A1		
chr2.0014931410	0.65	4.95E-02			
chr18.0047812935	-0.87	4.95E-02	CXXC1		
chr3.0049314217	-1.55	4.99E-02	C3orf62		
chr3.0076990952	-0.93	4.99E-02	ROBO2		X
chr15.0092571606	0.83	5.00E-02	SLCO3A1		

Table S3. Significant CpGs in MTs. LogFC = $\log_2$ fold change; positive logFC = hypermethylated in CP; negative logFC = hypomethylated in CP; blank values for gene = intergenic regions. X's indicate sites within muscle genes or annotated enhancers.

CpG site	LogFC	FDR corrected p-value	Gene	Muscle Gene	Enhancer Region
chr20.0054764055	-1.77	7.99E-19			
chr22.0041683914	-1.64	9.61E-18			
chr12.0128167651	1.57	7.53E-14			
chr15.0022731573	-3.11	8.31E-13			
chr11.0093971657	1.48	3.24E-12	RP11-680H20.2		
chr3.0051997994	2.37	3.48E-12	PCBP4, RP11-155D18.14, RP11-155D18.12		
chr20.0022553062	-2.00	8.86E-12	LINC00261		
chr20.0060375363	-1.50	8.88E-12	CDH4		
chr12.0001742005	-1.59	6.83E-11	WNT5B		
chr12.0114885652	-2.71	1.19E-10			
chr19.0048673525	2.17	8.48E-10	LIG1		
chr16.0058069921	1.35	2.56E-09	MMP15		
chr18.0071801714	1.29	5.18E-09	FBXO15		
chr15.0092982723	1.73	7.98E-09	ST8SIA2		
chr16.0002176197	2.29	3.62E-08	PKD1		
chr14.0019735451	-2.07	3.75E-08			
chr3.0073186712	1.17	6.27E-08			
chr11.0017593831	-1.48	8.49E-08	OTOG		
chr14.0035358595	1.17	1.09E-07			
chr4.0101719592	-1.15	1.50E-07	EMCN		
chr12.0031371137	1.21	2.28E-07			X
chr13.0089105339	1.17	2.85E-07			
chr14.0101270192	-2.14	4.57E-07	MEG3		
chr10.0047273588	-1.21	4.65E-07			
chr13.0113650227	-1.14	5.29E-07	MCF2L		
chr19.0019007351	1.65	6.17E-07	CERS1		
chr18.0043923940	-1.73	6.64E-07	RNF165		
chr12.0119584195	1.10	8.08E-07	SRRM4		
chr2.0056193463	1.55	1.07E-06	RP11-481J13.1, AC011306.2		
chr12.0002513357	1.11	1.13E-06	CACNA1C	X	
chr13.0028891055	1.13	1.21E-06	FLT1		
chr22.0046279322	1.50	1.23E-06	WI2-85898F10.1		
chr11.0123045794	-1.54	1.82E-06	CLMP		
chr5.0145724640	1.54	2.32E-06	CTC-359M8.1		
chr9.0043084487	1.71	2.70E-06	ANKRD20A3		

chr15.0067989241	1.16	3.44E-06	MAP2K5		
chr9.0000109396	-1.44	4.07E-06			
chr15.0069824154	1.55	4.20E-06	RP11-279F6.1		
chr12.0116068191	-1.58	4.42E-06	RP11-1028N23.4		
chr12.0133301799	-1.93	4.50E-06			
chr18.0011747918	-1.85	4.52E-06	GNAL		
chr16.0073089847	-1.42	4.72E-06	ZFHX3	X	
chr16.0000680326	-1.17	4.84E-06	WFIKKN1		
chr17.0075149309	1.38	5.78E-06	SEC14L1		
chr11.0067044849	1.99	6.16E-06	ADRBK1		
chr14.0034393486	1.09	6.26E-06	EGLN3		
chr12.0001609391	1.45	6.57E-06			
chr22.0040113559	1.04	6.88E-06			X
chr14.0104820848	2.33	7.61E-06			
chr20.0007787573	-1.03	7.76E-06			
chr15.0090964193	1.56	7.81E-06	IQGAP1		
chr20.0048793087	-1.09	7.84E-06	RP11-112L6.4		
chr12.0011513104	-1.02	8.25E-06	PRB1		
chr11.0116578644	1.50	8.36E-06			
chr17.0078426681	1.30	8.42E-06			
chr2.0067681880	-1.03	8.71E-06			
chr5.0172124187	1.46	8.81E-06	CTB-79E8.2		
chr7.0016768868	-1.03	8.88E-06			
chr20.0019221684	1.06	8.90E-06	SLC24A3		
chr20.0019447811	1.06	8.91E-06	SLC24A3		
chr20.0035217888	0.99	8.95E-06	RP5-977B1.11, TGIF2, TGIF2-C20orf24		
chr2.0029850455	1.32	9.56E-06	ALK		
chr18.0077209053	-1.56	9.74E-06	NFATC1		
chr3.0119863345	1.48	1.03E-05			
chr20.0060893697	-1.03	1.04E-05	LAMA5	X	
chr11.0126004170	1.28	1.08E-05			
chr13.0113705362	-1.58	1.13E-05			
chr2.0239051141	1.69	1.24E-05	KLHL30		
chr22.0044736662	1.52	1.45E-05			
chr3.0017180369	1.02	1.96E-05			
chr14.0105643016	1.49	2.15E-05	RP11-44N21.4, NUDT14		
chr22.0040899482	-1.03	2.22E-05	MKL1		
chr13.0093896533	2.41	2.50E-05	GPC6		
chr14.0104781790	1.55	2.51E-05			
chr14.0065204158	1.00	2.53E-05	PLEKHG3		
chr3.0122711856	1.43	2.94E-05	SEMA5B		

chr18.0044547036	1.72	2.97E-05	KATNAL2		
chr16.0066879958	1.00	3.34E-05	NAE1, RP11-61A14.1, CA7		X
chr16.0079468883	1.28	3.34E-05			
chr20.0035872503	-1.23	3.37E-05			
chr11.0129750009	-1.00	3.39E-05	NFRKB		
chr12.0016644803	-1.03	3.43E-05	MGST1		
chr3.0130802181	1.32	3.81E-05	NEK11		
chr19.0011533307	0.96	3.82E-05	CCDC151		
chr18.0045011716	1.41	4.02E-05	CTD-2130O13.1		
chr18.0023390824	0.99	4.44E-05			
chr12.0131689822	1.74	4.47E-05	RP11-638F5.1		
chr16.0002004921	1.33	4.90E-05	RPL3L		
chr3.0117631830	1.07	4.91E-05	LSAMP, RP11-384F7.2		
chr3.0016939628	-0.76	4.97E-05	PLCL2		
chr22.0038022735	1.46	4.98E-05	GGA1		
chr12.0057608666	2.24	5.16E-05			
chr13.0085360140	1.03	5.19E-05	SCARF2		
chr22.0020779274	1.36	5.19E-05			
chr12.0129065948	-1.87	5.22E-05	TMEM132C		
chr12.0010243158	1.52	5.34E-05	CLEC1A		
chr12.0083436417	2.14	5.88E-05	TMTC2		
chr15.0101588506	1.04	6.02E-05	LRRK1, RP11-505E24.3		
chr18.0006284010	0.98	6.44E-05	L3MBTL4		
chr14.0021177142	-1.24	6.51E-05			
chr2.0071533997	1.62	6.62E-05	ZNF638		X
chr14.0093019588	1.53	6.67E-05	RIN3		
chr4.0095555981	0.90	7.15E-05	PDLIM5	X	
chr3.0138666240	-1.23	7.17E-05	C3orf72		
chr16.0084054247	-1.12	7.23E-05	SLC38A8		
chr20.0061411239	1.36	7.28E-05			
chr2.0035092870	-1.19	7.35E-05	AC012593.1		
chr15.0098537476	1.15	7.59E-05			
chr20.0025001878	-1.34	8.18E-05	ACSS1		
chr16.0003238806	1.52	8.36E-05	AJ003147.9		
chr6.0016145414	-1.24	8.39E-05	MYLIP		
chr12.0101447687	1.57	8.98E-05	ANO4		
chr20.0056608905	1.00	8.98E-05			
chr10.0133837347	-1.19	9.10E-05			
chr14.0045546460	0.95	9.25E-05			
chr5.0177181499	-1.23	9.62E-05	FAM153A		
chr11.0129565594	1.68	1.09E-04			

chr15.0067076049	-0.95	1.13E-04			
chr20.0061921394	0.99	1.16E-04			
chr14.0069861077	-0.96	1.20E-04	ERH		
chr14.0096653669	-1.43	1.20E-04			X
chr3.0013245073	0.96	1.21E-04			
chr19.0002863955	1.17	1.22E-04			
chr12.0103344694	-1.28	1.22E-04	PAH		
chr20.0055369320	-1.68	1.23E-04			
chr9.0008638390	0.93	1.39E-04	PTPRD		
chr6.0159600100	1.37	1.40E-04	FNDC1		X
chr13.0044362688	1.04	1.42E-04			
chr9.0026632294	0.92	1.45E-04			
chr19.0007038916	-1.20	1.51E-04	MBD3L4		
chr17.0036292033	2.25	1.66E-04	TBC1D3F		
chr15.0069452943	1.57	1.66E-04	GLCE		
chr17.0073069667	0.92	1.66E-04			
chr5.0177219938	-0.97	1.75E-04	RP11-1026M7.2		
chr19.0008750022	1.20	1.82E-04			
chr14.0080449863	-1.74	1.82E-04			X
chr2.0023747265	1.15	1.82E-04	KLHL29		
chr14.0021316565	-1.56	1.82E-04			
chr17.0018941025	-1.96	1.85E-04	GRAP		
chr14.0053155967	-0.96	1.86E-04	ERO1L		
chr5.0164550214	-1.19	2.14E-04	CTC-340A15.2		
chr5.0122422526	1.39	2.21E-04			
chr17.0038475164	-1.29	2.26E-04	RARA		
chr18.0029197908	1.13	2.30E-04			
chr14.0104190006	-1.75	2.35E-04	ZFYVE21		
chr12.0130645000	-1.81	2.38E-04	FZD10-AS1		
chr20.0059927068	0.96	2.48E-04			
chr5.0106482812	-1.22	2.57E-04			
chr6.0164766175	-1.55	2.58E-04			
chr14.0101909600	1.07	2.75E-04	RP11-168L7.3		X
chr18.0076123813	-1.22	2.79E-04			
chr17.0079292270	0.91	2.87E-04	TMEM105		
chr14.0104465222	0.88	2.88E-04	TDRD9		
chr19.0016830739	1.11	3.11E-04			
chr15.0069516868	1.65	3.22E-04			
chr17.0074566299	1.14	3.38E-04	ST6GALNAC2, RP11-666A8.9		
chr12.0006104973	-1.64	3.48E-04	VWF		
chr3.0189596966	-1.29	3.62E-04	TP63		

chr2.0218771815	-1.20	3.62E-04	TNS1		
chr15.0058213431	1.26	3.68E-04			X
chr15.0057921004	0.93	3.75E-04	GCOM1, MYZAP, POLR2M		
chr14.0069738172	-1.25	3.87E-04	GALNT16		X
chr15.0045573050	-1.11	3.95E-04			
chr19.0004596412	1.20	4.13E-04			
chr17.0053343752	1.76	4.27E-04	HLF	X	
chr2.0238831642	1.32	4.33E-04			
chr5.0166472226	-1.16	4.40E-04			
chr12.0085129789	1.31	4.59E-04			
chr9.0035610018	0.86	4.59E-04	TESK1, CD72		
chr17.0002701265	1.19	4.64E-04	RAP1GAP2		
chr15.0055457141	1.19	4.75E-04			
chr5.0131564700	-1.05	4.76E-04	P4HA2		
chr2.0107178887	1.28	4.77E-04			
chr12.0101846506	1.04	4.81E-04			
chr9.0120247630	-1.36	4.81E-04			X
chr13.0027424109	1.56	4.94E-04			X
chr6.0151563368	-0.67	5.08E-04	AKAP12		
chr22.0022129567	1.57	5.35E-04	MAPK1		
chr2.0060196436	0.91	5.36E-04	RP11-444A22.1		
chr2.0129148635	0.93	5.37E-04			
chr20.0048993491	-0.65	5.39E-04			
chr14.0061281110	0.88	5.55E-04	MNAT1		
chr3.0149299399	1.18	5.63E-04	WWTR1		
chr2.0235215325	-1.08	5.88E-04			
chr9.0044359093	-1.56	5.99E-04			
chr13.0042220397	-1.10	6.05E-04	VWA8		
chr4.0174264841	0.88	6.07E-04			
chr12.0114238657	0.89	6.08E-04			
chr19.0039524206	-1.08	6.25E-04			
chr10.0010604528	0.99	6.32E-04			
chr15.0068639973	1.53	6.66E-04	ITGA11	X	
chr19.0045735345	-1.09	6.74E-04	MARK4, EXOC3L2		
chr2.0113399982	0.98	6.75E-04	AC079922.3		
chr16.0002478353	-1.36	7.18E-04			
chr17.0077893455	1.55	7.25E-04	RP11-353N14.5, RP11-353N14.4		
chr17.0019045779	-1.56	7.25E-04	GRAPL, CTC-457L16.2		
chr13.0052817715	-1.01	7.59E-04	RP11-248G5.8, TPTE2P2		
chr20.0059339955	1.00	7.92E-04			
chr11.0046135102	0.86	7.96E-04	PHF21A		

chr20.0060885977	-0.90	7.98E-04			
chr10.0096151033	-0.84	7.99E-04			
chr15.0078689511	1.84	8.13E-04			X
chr3.0053784559	1.14	8.13E-04	CACNA1D	X	
chr15.0043244495	-0.88	8.15E-04	UBR1		
chr22.0029463592	-1.23	8.18E-04			
chr13.0102572261	-1.39	8.21E-04	FGF14		
chr22.0042875005	1.41	8.23E-04			X
chr16.0088038262	1.25	8.24E-04	BANP		
chr14.0104977792	1.66	8.42E-04	TMEM179		
chr19.0010249278	1.07	8.45E-04	DNMT1		
chr21.0044484874	1.12	8.48E-04	CBS		
chr20.0060501154	1.87	8.50E-04			
chr17.0026101385	0.88	8.55E-04	NOS2		
chr16.0016763207	-1.39	8.57E-04			
chr17.0030126050	-1.04	8.63E-04			
chr12.0052817612	1.90	8.70E-04	RP11-1020M18.10		
chr14.0096628985	1.05	9.38E-04			
chr15.0034647112	-0.86	9.41E-04	NUTM1		
chr6.0030746896	1.34	9.42E-04	HCG20		
chr5.0164483805	-1.11	9.49E-04			
chr15.0098986819	1.07	9.59E-04	FAM169B		X
chr12.0033043614	0.91	1.00E-03	PKP2	X	
chr10.0105420300	-1.73	1.03E-03	SH3PXD2A		
chr21.0030689317	-0.86	1.05E-03	BACH1		
chr14.0043795919	-1.01	1.06E-03			
chr11.0068950670	1.04	1.07E-03			
chr6.0117187287	-0.97	1.08E-03			
chr14.0093530974	1.55	1.12E-03	ITPK1		
chr14.0105065084	2.21	1.12E-03			X
chr14.0100040905	-1.28	1.16E-03	CCDC85C		
chr14.0055966140	1.12	1.17E-03	KTN1-AS1		X
chr17.0019046425	-1.38	1.19E-03			
chr22.0050332646	-1.35	1.20E-03			
chr14.0075517195	1.29	1.21E-03	MLH3		
chr15.0088659150	1.60	1.21E-03	NTRK3		X
chr2.0046561399	-1.02	1.22E-03	EPAS1		
chr20.0037906145	1.06	1.23E-03			
chr18.0072843785	-1.61	1.26E-03			
chr12.0052981404	1.00	1.26E-03	KRT72		
chr22.0030084328	0.88	1.28E-03	NF2		

chr13.0099764013	0.87	1.28E-03			
chr22.0050341737	-0.96	1.31E-03			
chr15.0033445994	0.87	1.32E-03	FMN1		
chr11.0042088046	0.89	1.33E-03	RP11-148I19.1		
chr12.0013354616	1.14	1.34E-03	EMP1		
chr17.0070499160	1.06	1.34E-03	LINC00511		
chr15.0080573863	0.88	1.35E-03	LINC00927		
chr10.0104354945	-0.83	1.35E-03	SUFU		
chr9.0018605667	-1.06	1.36E-03	ADAMTSL1		
chr14.0105040488	1.64	1.36E-03			
chr15.0101482307	-0.86	1.36E-03			
chr20.0060272050	0.98	1.36E-03			
chr10.0004224715	-1.04	1.36E-03			
chr6.0001385426	1.02	1.37E-03			
chr12.0010242982	1.25	1.38E-03			
chr11.0070369651	1.18	1.39E-03	SHANK2		
chr13.0077121220	1.42	1.40E-03			
chr12.0046149812	-0.98	1.44E-03	ARID2		
chr18.0046461592	1.01	1.44E-03	SMAD7	X	
chr13.0028302652	1.74	1.44E-03			X
chr13.0047916427	-0.93	1.44E-03			
chr22.0038010164	1.17	1.45E-03			
chr2.0237117273	-1.03	1.45E-03	AC079135.1, ASB18		
chr18.0038139510	1.29	1.45E-03			
chr3.0060920593	0.96	1.50E-03	FHIT		
chr17.0065622780	-0.86	1.53E-03	PITPNC1		
chr8.0042999184	-0.83	1.55E-03	HGSNAT		
chr14.0021179750	-1.08	1.56E-03			
chr9.0116266123	0.90	1.56E-03	RGS3		
chr5.0137549862	1.24	1.63E-03			
chr12.0122395466	-0.85	1.63E-03	WDR66		
chr12.0120463149	-0.88	1.63E-03	CCDC64		
chr18.0071529044	1.14	1.64E-03			
chr13.0045890247	1.04	1.70E-03			
chr9.0138653925	0.83	1.84E-03	KCNT1		
chr16.0000555577	-0.82	1.91E-03	RAB11FIP3		
chr13.0113678380	-1.34	1.92E-03			
chr12.0063544384	-1.06	1.94E-03	AVPR1A		
chr3.0077077670	0.84	1.95E-03	ROBO2		
chr22.0020244288	-1.28	1.97E-03	RTN4R		
chr13.0033220266	-1.31	1.97E-03	PDS5B		

chr6.0021679242	1.01	2.00E-03	CASC15		
chr16.0077912976	-1.28	2.00E-03	VAT1L		
chr20.0048859896	1.74	2.05E-03			
chr17.0008238137	-1.14	2.05E-03			
chr12.0053189775	1.06	2.05E-03	KRT3		
chr14.0096729260	1.15	2.08E-03	RP11-404P21.8, BDKRB1, RP11-404P21.3		
chr2.0047454097	-0.94	2.22E-03	AC073283.4, AC106869.2		
chr3.0111469507	-1.01	2.23E-03	PLCXD2, PHLDB2		
chr12.0066791524	1.02	2.23E-03	GRIP1		
chr18.0020972062	-0.89	2.25E-03	TMEM241		
chr13.0074262821	-0.94	2.25E-03	KLF12		
chr15.0088174247	1.02	2.26E-03	RP11-648K4.2		
chr22.0049403377	1.35	2.27E-03			
chr6.0008948266	1.24	2.27E-03			
chr5.0159719484	0.82	2.28E-03	CCNJL		
chr14.0075019216	-1.30	2.28E-03	LTBP2, CTD-2207P18.1		
chr8.0001828441	1.25	2.29E-03	ARHGEF10		
chr12.0106248941	1.04	2.30E-03			
chr3.0167620956	-0.90	2.31E-03	RP11-298O21.5		
chr10.0126136854	1.31	2.32E-03	NKX1-2, RP13-238F13.3		
chr19.0029574847	-1.29	2.35E-03			
chr22.0019946864	1.15	2.38E-03	COMT		
chr2.0111241804	1.04	2.44E-03			
chr11.0001418756	1.15	2.51E-03	BRSK2		
chr17.0031110221	-1.32	2.51E-03	MYO1D		
chr16.0001339089	1.38	2.66E-03			
chr20.0036515329	1.07	2.67E-03			X
chr14.0050108699	1.49	2.69E-03			
chr16.0083715823	1.18	2.80E-03	CDH13		X
chr14.0085404000	-1.38	2.81E-03			
chr15.0101984558	1.12	2.82E-03	PCSK6		
chr11.0062211039	1.01	2.84E-03	AHNAK		
chr10.0068984115	0.79	2.85E-03	CTNNA3		
chr15.0029644298	0.97	2.85E-03	FAM189A1		
chr14.0094578378	1.16	2.85E-03	IFI27		
chr6.0044243576	1.12	2.86E-03	TMEM151B, RP11-444E17.6		
chr20.0052825772	-1.31	2.86E-03	PFDN4		
chr17.0000112167	0.83	2.86E-03	RPH3AL		
chr17.0026875323	1.24	2.86E-03	RP11-192H23.4, UNC119		
chr3.0016419520	1.28	2.88E-03	RFTN1		
chr11.0065261151	1.54	2.90E-03			

chr22.0033976935	0.98	2.91E-03	LARGE1	X	
chr22.0031890427	-1.19	2.92E-03	DRG1, EIF4ENIF1, SFI1		
chr5.0003819923	0.82	2.94E-03			
chr22.0029704946	1.44	2.95E-03	GAS2L1		
chr18.0009870097	-1.48	2.95E-03			
chr18.0075321554	1.22	2.95E-03			
chr12.0113977822	0.87	2.96E-03			
chr2.0239196855	-1.12	2.96E-03	PER2		
chr22.0046472984	-1.35	2.96E-03	FLJ27365		
chr12.0123780584	-0.81	2.97E-03	SBNO1		
chr12.0005898164	1.03	2.98E-03	ANO2		
chr2.0086148506	1.07	2.98E-03			
chr5.0087955859	-1.35	3.03E-03	LINC00461		
chr14.0089771498	1.60	3.17E-03	FOXN3		
chr22.0033739403	0.82	3.17E-03			
chr18.0047230566	-1.30	3.20E-03			X
chr5.0092767677	-0.87	3.21E-03	NR2F1-AS1		
chr3.0123171493	-0.89	3.27E-03			
chr18.0011426911	-1.01	3.28E-03			
chr5.0176220557	1.23	3.29E-03			X
chr2.0182549408	-1.34	3.30E-03	AC013733.3		
chr11.0122674083	1.16	3.31E-03	UBASH3B		
chr6.0154640863	1.34	3.35E-03	IPCEF1		
chr3.0045798106	0.81	3.38E-03	SLC6A20		
chr12.0131588481	1.10	3.43E-03	GPR133		
chr12.0057602992	1.61	3.48E-03	LRP1		
chr2.0020252403	-1.22	3.48E-03	RP11-644K8.1		
chr3.0054664460	0.95	3.53E-03	CACNA2D3		
chr16.0031091597	-0.81	3.53E-03	ZNF646		
chr2.0114025250	0.90	3.59E-03	PAX8, PAX8-AS1		
chr19.0009956949	-0.82	3.62E-03	PIN1		
chr13.0036668831	1.14	3.63E-03	DCLK1		
chr20.0015883372	0.83	3.63E-03	MACROD2		
chr12.0081306216	0.93	3.64E-03	LIN7A		
chr13.0020646254	0.93	3.64E-03	ZMYM2, KRR1P1		
chr3.0160006045	0.95	3.65E-03	RP11-432B6.3, IFT80		
chr11.0002408146	1.41	3.65E-03	CD81		
chr17.0043201589	1.33	3.66E-03	PLCD3		
chr18.0011979862	-0.85	3.71E-03			
chr17.0047518498	0.87	3.73E-03	RP11-81K2.1		
chr19.0046730089	1.06	3.77E-03			X

chr16.0087893023	-1.32	3.79E-03	SLC7A5		
chr11.0000766885	-1.25	3.79E-03			X
chr12.0117176320	1.99	3.83E-03	RNFT2		
chr15.0057680681	-1.25	3.87E-03	CGNL1		
chr15.0081187412	0.97	3.92E-03	KIAA1199		
chr11.0047295306	0.95	3.98E-03	MADD		
chr14.0038781301	1.00	3.99E-03			
chr2.0221113349	0.84	4.07E-03	AC114765.1		
chr16.0007479261	-1.05	4.11E-03	RBFOX1		
chr11.0017601401	-0.95	4.14E-03			
chr12.0039057639	0.92	4.16E-03	CPNE8		
chr14.0073050565	1.25	4.17E-03	RP3-514A23.2		
chr11.0116705996	1.27	4.31E-03			
chr20.0030285535	-0.97	4.36E-03	BCL2L1, RP11-243J16.7		
chr20.0031275087	-0.85	4.36E-03			
chr17.0028803808	-1.24	4.38E-03			
chr11.0002800586	1.27	4.39E-03	KCNQ1	X	
chr20.0031490465	-1.45	4.40E-03	EFCAB8		X
chr11.0070397632	1.18	4.40E-03			X
chr2.0064955687	0.78	4.41E-03	SERTAD2		
chr19.0052801607	-0.93	4.41E-03	ZNF480		
chr22.0033791071	0.84	4.42E-03			
chr12.0105913687	1.02	4.48E-03			
chr19.0041126191	-0.92	4.48E-03	LTBP4		
chr11.0032197363	0.80	4.49E-03	RP1-65P5.1		
chr15.0088159831	0.98	4.49E-03			
chr16.0070746421	-0.92	4.50E-03	VAC14		
chr10.0017068488	0.79	4.51E-03	CUBN		
chr18.0053091201	-1.11	4.53E-03	TCF4		
chr17.0079501119	1.48	4.53E-03	FSCN2		X
chr17.0076513353	-0.80	4.54E-03	DNAH17		
chr20.0057966327	-1.33	4.56E-03			
chr12.0104187430	0.94	4.68E-03	NT5DC3		
chr22.0018433398	-1.70	4.71E-03	MICAL3		
chr5.0011535023	1.19	4.71E-03	CTNND2		
chr9.0000947870	0.83	4.71E-03	DMRT1		
chr6.0007765009	0.83	4.72E-03	BMP6		
chr13.0115035743	-0.80	4.72E-03	CDC16		
chr17.0046667531	0.93	4.73E-03	HOXB-AS3, HOXB3		
chr3.0182124231	-1.15	4.74E-03			X
chr15.0083088304	0.83	4.74E-03	RPL9P8, UBE2Q2P3, RP11-152F13.8		

chr15.0031423000	-1.23	4.75E-03	TRPM1		
chr5.0174109443	-0.81	4.75E-03			
chr18.0030519743	-1.02	4.76E-03	CCDC178		X
chr12.0066354814	0.99	4.77E-03	HMGA2		
chr20.0043344103	1.32	4.77E-03	RP11-445H22.4, WISP2		
chr2.0111791669	0.81	4.83E-03	ACOXL		
chr6.0170373616	-0.91	4.90E-03			
chr14.0077057981	-1.15	4.94E-03	RP11-187O7.3		
chr2.0185490688	0.78	4.95E-03	ZNF804A		
chr16.0062003103	-1.29	4.96E-03	CDH8		
chr7.0001278669	-1.17	5.00E-03			
chr5.0111578627	0.83	5.02E-03	EPB41L4A, RP11-526F3.1		
chr14.0074764684	-0.90	5.05E-03	ABCD4		
chr16.0048419832	-1.42	5.06E-03	SIAH1		
chr16.0090008697	-0.87	5.06E-03			
chr6.0001768121	1.29	5.07E-03	GMDS		
chr5.0075360503	-1.03	5.13E-03			
chr12.0132130323	2.53	5.14E-03			X
chr12.0000566229	0.92	5.14E-03			
chr12.0086359099	0.96	5.15E-03			
chr6.0011824090	0.80	5.18E-03			
chr3.0005182629	-0.87	5.19E-03	ARL8B		
chr16.0071043770	-1.00	5.22E-03	HYDIN		
chr14.0099456733	0.82	5.23E-03			
chr14.0105668565	-1.17	5.23E-03			
chr6.0075912800	1.03	5.24E-03	COL12A1		
chr15.0061121482	0.96	5.33E-03	RORA		
chr9.0084208113	0.92	5.35E-03	TLE1		
chr15.0051977900	0.84	5.38E-03	SCG3		
chr6.0004100037	0.92	5.38E-03	C6orf201		
chr15.0082092159	1.04	5.39E-03	RP11-499F3.2		
chr15.0051280672	-0.87	5.40E-03	AP4E1		
chr15.0043969272	0.87	5.50E-03	STRC, AC011330.5		
chr14.0105285074	0.99	5.50E-03			
chr15.0071044501	0.99	5.53E-03	UACA, RP11-138H8.2		
chr12.0001241667	-0.57	5.65E-03	ERC1		
chr14.0050108792	1.35	5.68E-03			
chr13.0109856377	-1.52	5.71E-03	MYO16		X
chr12.0132249669	0.81	5.73E-03	SFSWAP		
chr3.0109036950	0.90	5.73E-03			
chr15.0052549111	1.07	5.80E-03	MYO5C		

chr9.0000286627	0.89	5.80E-03	DOCK8		
chr22.0038696824	-0.77	5.80E-03	CSNK1E		
chr8.0001112153	0.74	5.82E-03	CTD-2281E23.2		
chr20.0048363935	-0.66	5.83E-03			
chr5.0011632009	0.92	5.83E-03			
chr18.0074616984	1.08	5.83E-03	ZNF236		
chr2.0015301469	0.93	5.84E-03			
chr13.0047191668	-1.34	5.86E-03	LRCH1		
chr6.0071209322	0.89	5.87E-03	FAM135A		
chr3.0127606140	-1.12	5.88E-03			
chr18.0007170034	-1.66	5.89E-03			
chr6.0052861066	1.30	5.89E-03			
chr15.0078008856	1.04	5.90E-03	LINGO1		
chr20.0018295860	0.86	5.97E-03	ZNF133		
chr12.0003242436	1.10	5.99E-03	TSPAN9		
chr14.0094215266	1.00	5.99E-03	PRIMA1		
chr14.0034616607	-1.10	6.00E-03			
chr3.0009152200	-1.21	6.03E-03	SRGAP3		
chr12.0128790228	-1.16	6.04E-03			
chr5.0149438902	0.76	6.10E-03	CSF1R		
chr12.0023768031	-0.90	6.13E-03	SOX5		
chr14.0047624779	0.90	6.17E-03	MDGA2		
chr10.0036627478	-0.79	6.24E-03			
chr18.0024443615	-1.22	6.29E-03	AQP4-AS1, AQP4		
chr2.0010545065	-1.17	6.35E-03	HPCAL1		
chr18.0042403544	1.01	6.36E-03	SETBP1		X
chr17.0011390406	1.32	6.37E-03	SHISA6		X
chr20.0006327945	0.95	6.43E-03			
chr12.0126468360	-1.52	6.46E-03	RP5-916L7.2		
chr11.0092261532	1.02	6.47E-03	FAT3		
chr14.0106416958	1.44	6.58E-03			
chr11.0002911200	0.93	6.58E-03	SLC22A18AS		
chr15.0039617456	0.97	6.59E-03	RP11-624L4.1		
chr14.0025947530	1.05	6.61E-03			
chr2.0038389310	1.15	6.61E-03	CYP1B1-AS1		
chr22.0039605885	0.86	6.63E-03			
chr6.0044242577	0.87	6.66E-03			
chr12.0000313066	1.52	6.88E-03	SLC6A12, RP11-283I3.2		
chr21.0046032860	0.97	6.91E-03	TSPEAR, KRTAP10-8		
chr16.0082973314	0.79	6.93E-03			
chr15.0060047878	1.04	6.96E-03			

chr12.0067447796	0.98	7.08E-03	RP11-123O10.4, RP11-123O10.3		
chr12.0052969844	0.91	7.12E-03			
chr5.0011534641	1.05	7.25E-03			
chr3.0152680039	1.12	7.26E-03			
chr20.0045860407	0.99	7.36E-03	ZMYND8		
chr22.0019036590	-0.94	7.37E-03	DGCR2		
chr16.0081443989	0.78	7.37E-03			
chr13.0100228957	-0.89	7.38E-03			
chr18.0018606618	-0.91	7.40E-03	ROCK1	X	
chr12.0053958599	0.98	7.41E-03	RP11-793H13.10, ATF7		
chr3.0109858228	1.23	7.41E-03			
chr2.0242709430	1.03	7.42E-03			
chr15.0042067504	1.56	7.45E-03	MAPKBP1		
chr12.0098384560	0.94	7.49E-03			
chr2.0023747257	0.84	7.50E-03			
chr7.0056844460	0.84	7.58E-03			
chr9.0042897524	1.11	7.65E-03			
chr12.0095551716	-0.89	7.77E-03	FGD6		
chr6.0051247041	0.80	7.77E-03			
chr14.0078559726	0.96	7.81E-03			
chr20.0019488775	1.08	7.81E-03			
chr20.0019419638	-1.61	7.82E-03			
chr20.0060375820	-1.58	7.85E-03			
chr11.0009068783	1.08	7.89E-03	RP11-467K18.2, SCUBE2		
chr18.0047177650	-0.64	7.90E-03			X
chr7.0140039833	0.72	7.94E-03	SLC37A3		
chr20.0025513653	1.09	7.95E-03	NINL		
chr5.0071385502	-0.78	8.04E-03			
chr16.0070734806	1.35	8.06E-03			
chr15.0079070483	-1.15	8.06E-03	ADAMTS7		
chr15.0028343785	-2.11	8.07E-03	OCA2		
chr3.0023957967	2.03	8.12E-03	NKIRAS1		
chr2.0005679673	0.99	8.14E-03			
chr12.0110563292	-1.13	8.16E-03	IFT81		
chr12.0005678978	1.08	8.20E-03			
chr3.0071153921	0.89	8.21E-03	FOXP1		
chr12.0005275433	0.95	8.21E-03			
chr7.0075004051	-1.06	8.21E-03	STAG3L1, AC006014.1		
chr14.0051538766	1.36	8.22E-03	TRIM9		
chr17.0073837683	0.73	8.26E-03	UNC13D		
chr18.0040318937	-0.99	8.27E-03			

chr7.0074333014	-0.82	8.28E-03	PMS2P5		
chr16.0016734442	-0.92	8.36E-03			
chr14.0095999996	-0.98	8.36E-03	SNHG10, GLRX5		
chr6.0027950800	0.81	8.39E-03			
chr13.0082065577	-0.88	8.41E-03			
chr20.0036854808	0.88	8.42E-03	KIAA1755		
chr11.0067049045	-0.78	8.42E-03			
chr15.0032004823	-0.89	8.43E-03	OTUD7A		
chr5.0050585672	0.78	8.43E-03			
chr12.0131392155	-0.95	8.56E-03			
chr17.0042636770	0.89	8.56E-03	FZD2	X	
chr20.0017403368	-1.05	8.58E-03	PCSK2		
chr16.0004815786	-0.92	8.61E-03	ZNF500		
chr12.0000650002	0.80	8.61E-03	B4GALNT3		
chr16.0001334537	0.98	8.66E-03			
chr13.0026651855	0.91	8.66E-03			
chr17.0064774558	0.74	8.75E-03	PRKCA		
chr2.0119616334	0.85	8.76E-03			
chr8.0135941900	0.92	8.77E-03			
chr5.0083244775	0.77	8.77E-03	EDIL3		
chr22.0025563508	0.98	8.79E-03	KIAA1671		X
chr17.0034406350	-0.90	8.80E-03	AC069363.1		
chr17.0046524375	0.92	8.85E-03			
chr6.0074643904	-0.86	8.86E-03			
chr18.0046475320	-1.11	8.87E-03			
chr20.0047278938	1.01	8.94E-03	PREX1		X
chr11.0035250260	0.88	8.96E-03	CD44		
chr12.0002398915	-0.68	8.97E-03			
chr2.0020378233	-1.01	8.98E-03			
chr14.0053315566	1.08	9.10E-03			
chr3.0035240556	0.91	9.11E-03			
chr15.0069894932	0.89	9.14E-03	RP11-279F6.3		
chr3.0139013512	0.77	9.22E-03	MRPS22		
chr13.0095256511	-1.49	9.26E-03	GPR180		
chr20.0044107407	0.90	9.27E-03	AL031663.1, WFDC2		
chr17.0049983647	0.86	9.27E-03	CA10		
chr13.0030932893	1.02	9.29E-03	LINC00426		
chr11.0120972213	0.86	9.30E-03	TECTA		X
chr12.0019243112	0.92	9.50E-03			
chr12.0066742963	0.91	9.53E-03			
chr20.0021535931	1.19	9.62E-03			X

chr19.0050184400	-0.89	9.62E-03	PRMT1		
chr6.0111244762	0.79	9.65E-03			
chr2.0001598523	-1.06	9.74E-03	AC144450.1		
chr14.0057436305	0.87	9.75E-03	OTX2-AS1		
chr3.0026305995	0.86	9.77E-03			
chr17.0078441727	0.86	9.79E-03	NPTX1		
chr3.0132565636	0.80	9.82E-03	NPHP3-AS1		
chr16.0088184978	1.18	9.82E-03			
chr12.0039484618	-0.88	9.83E-03	RP11-554L12.1		X
chr2.0207422413	0.81	9.87E-03	ADAM23		
chr11.0112152163	-0.94	9.88E-03	RP11-356J5.12		
chr12.0095411841	-0.93	9.91E-03			
chr22.0038573766	1.39	9.96E-03	PLA2G6	X	
chr12.0102034085	0.71	9.97E-03	MYBPC1	X	
chr2.0075449181	-1.09	1.00E-02			X
chr14.0089833851	1.32	1.01E-02			
chr10.0091818139	-0.91	1.01E-02			
chr12.0103102106	1.00	1.02E-02			
chr5.0128430539	1.21	1.03E-02	ISOC1		
chr3.0053806824	1.12	1.04E-02			
chr12.0120234027	0.94	1.04E-02	CIT		
chr13.0111606387	-0.88	1.04E-02			
chr18.0021691205	-0.87	1.05E-02	TTC39C		
chr3.0083809794	0.82	1.05E-02			
chr13.0114825688	-1.31	1.05E-02	RASA3		
chr20.0040847575	1.27	1.05E-02	PTPRT		
chr18.0043301302	0.93	1.06E-02	RP11-116O18.3		
chr12.0122768292	-0.94	1.06E-02	CLIP1		
chr3.0047630484	0.84	1.06E-02	SMARCC1		
chr13.0047010408	-0.86	1.06E-02	PPP1R2P4, KIAA0226L		
chr13.0019443315	-1.11	1.06E-02	ANKRD20A9P		
chr11.0116340736	0.84	1.06E-02			
chr17.0031370105	0.86	1.06E-02	ASIC2		
chr8.0086566777	0.97	1.06E-02			
chr11.0076392234	1.23	1.06E-02	RP11-672A2.3		
chr5.0106822941	-1.17	1.06E-02	EFNA5		
chr20.0056321421	0.85	1.06E-02			
chr12.0085198936	-0.84	1.06E-02			
chr15.0089869670	-0.89	1.06E-02	POLG		
chr16.0078800215	0.93	1.07E-02	WWOX		
chr20.0062445219	-0.90	1.07E-02	ZBTB46, RP4-583P15.11		

chr7.0042657968	-1.04	1.11E-02			
chr12.0112591200	-0.75	1.11E-02	TRAFD1		
chr4.0041302228	0.74	1.11E-02			
chr16.0057821860	0.81	1.11E-02	KIFC3		
chr11.0099244166	0.71	1.11E-02	CNTN5		
chr2.0223550141	-1.08	1.11E-02	MOGAT1		
chr22.0018376899	0.75	1.11E-02			
chr10.0080833721	-1.11	1.13E-02	ZMIZ1		
chr3.0108773581	-0.77	1.13E-02	MORC1		
chr12.0008403064	-0.99	1.13E-02			
chr20.0036843383	0.98	1.13E-02			
chr6.0006687652	1.08	1.14E-02			
chr6.0000281849	0.84	1.14E-02			
chr12.0054043247	-1.13	1.14E-02	ATP5G2		
chr20.0001412499	-0.84	1.15E-02			
chr20.0048603774	0.90	1.15E-02	SNAI1		
chr19.0047299878	0.91	1.16E-02			
chr5.0174825487	1.19	1.17E-02			
chr15.0068885184	0.88	1.17E-02	CORO2B		X
chr9.0138974172	-0.90	1.17E-02	NACC2		
chr20.0043391328	1.32	1.17E-02	RIMS4		
chr12.0025101805	1.72	1.17E-02	BCAT1		
chr14.0020089147	-1.69	1.17E-02	RP11-597A11.1		
chr1.0032307779	-0.83	1.18E-02	RP11-84A19.2		
chr17.0034580968	1.69	1.19E-02	TBC1D3C		
chr6.0140996256	0.82	1.19E-02			
chr14.0059509566	-1.00	1.19E-02			
chr13.0037937255	-0.92	1.19E-02			
chr15.0079292108	0.85	1.20E-02	RASGRF1		X
chr13.0107176083	-1.68	1.20E-02	EFNB2		
chr15.0070210541	0.99	1.20E-02			
chr2.0010200647	1.30	1.21E-02	CYS1		
chr7.0025548858	0.75	1.22E-02			
chr20.0052298155	-0.85	1.22E-02			
chr20.0060928261	-0.97	1.23E-02	RP11-157P1.5		
chr6.0166756539	-1.15	1.23E-02			
chr20.0036859462	0.89	1.23E-02			
chr13.0021819967	1.03	1.24E-02			
chr2.0209313215	-0.83	1.24E-02	PTH2R		
chr12.0114887143	0.96	1.25E-02			
chr15.0050873417	0.87	1.25E-02	TRPM7		

chr6.0008223055	0.86	1.26E-02			
chr16.0011058849	-0.82	1.26E-02	CLEC16A		
chr7.0044807421	1.17	1.26E-02	ZMIZ2		
chr15.0066557418	-1.14	1.27E-02			
chr14.0061919248	-0.88	1.28E-02	PRKCH		
chr6.0015748745	0.85	1.28E-02			
chr12.0130090963	0.92	1.28E-02	TMEM132D		
chr15.0078076885	0.71	1.28E-02			
chr13.0100623759	1.33	1.28E-02	ZIC5		
chr12.0124860589	0.99	1.29E-02	NCOR2		
chr2.0062702536	1.09	1.29E-02			
chr14.0094629010	0.86	1.29E-02	PPP4R4		
chr10.0097051250	0.90	1.29E-02			
chr13.0099413484	-0.83	1.29E-02			
chr13.0033765016	-1.51	1.30E-02	STARD13		
chr16.0089069833	1.03	1.30E-02			X
chr11.0063901203	0.83	1.30E-02	MACROD1		
chr11.0000597676	-0.74	1.30E-02	PHRF1		
chr9.0079793512	1.19	1.31E-02	VPS13A		
chr20.0038382953	-0.98	1.32E-02			
chr13.0104109509	0.87	1.33E-02			
chr15.0040545511	1.20	1.33E-02	RP11-133K1.2, PAK6		
chr3.0178199704	0.83	1.33E-02	KCNMB2		
chr16.0029168316	1.09	1.33E-02	RP11-426C22.5		
chr6.0127687678	1.38	1.33E-02			
chr11.0070384416	1.39	1.34E-02			
chr3.0099514929	-0.84	1.34E-02	COL8A1		
chr3.0196595774	-1.33	1.34E-02	SENP5		
chr6.0073290272	1.30	1.34E-02			
chr19.0009468870	0.74	1.36E-02	ZNF559-ZNF177, ZNF177		
chr2.0204746463	-1.07	1.37E-02			
chr14.0039235379	1.02	1.37E-02	LINC00639		
chr3.0125867487	0.78	1.37E-02	ALDH1L1		
chr15.0051183110	1.16	1.38E-02			
chr11.0115343899	0.82	1.38E-02	CADM1		
chr3.0056912875	0.84	1.38E-02	ARHGEF3		
chr12.0056810802	-0.86	1.38E-02			
chr11.0046483909	0.74	1.39E-02	AMBRA1		X
chr5.0143251840	-0.96	1.39E-02			
chr17.0080152801	-0.75	1.39E-02	CCDC57		
chr3.0075160661	0.82	1.39E-02			

chr20.0024775451	1.04	1.39E-02			
chr14.0096856226	-0.83	1.39E-02			
chr12.0109897832	0.79	1.42E-02	KCTD10		
chr18.0077608332	1.01	1.42E-02			
chr18.0023185698	-1.22	1.43E-02			X
chr14.0049874442	-0.87	1.43E-02			
chr14.0105753103	-1.05	1.43E-02	BRF1		
chr2.0069171202	1.37	1.43E-02			
chr17.0011356330	-1.04	1.44E-02			
chr3.0037374195	0.73	1.44E-02	GOLGA4		
chr12.0113335076	-1.19	1.44E-02	RPH3A		
chr3.0059224627	0.74	1.44E-02			
chr8.0001251943	0.71	1.44E-02			
chr3.0180905890	-0.73	1.44E-02	SOX2-OT		
chr6.0031008851	1.01	1.45E-02			
chr12.0084953248	-0.83	1.45E-02			
chr20.0035201174	1.15	1.46E-02	RP5-977B1.7		
chr22.0049199174	1.15	1.46E-02	FAM19A5		
chr22.0028173173	0.98	1.48E-02	MN1		
chr12.0095457527	-1.05	1.48E-02	NR2C1		
chr6.0032026797	0.92	1.48E-02	TNXB		
chr2.0102423165	1.08	1.48E-02	MAP4K4		
chr18.0045776354	-0.50	1.48E-02	ZBTB7C		
chr20.0055621592	-1.35	1.48E-02			
chr14.0081456317	0.92	1.48E-02	TSHR		
chr8.0140921136	0.73	1.48E-02	TRAPPC9		
chr20.0058775716	1.03	1.48E-02	RP5-1043L13.1		
chr13.0036606864	1.01	1.50E-02			
chr5.0032690664	0.95	1.50E-02	NPR3		
chr13.0096130928	-1.29	1.50E-02	CLDN10		
chr2.0235873378	0.97	1.50E-02	SH3BP4		
chr20.0003316125	-0.87	1.50E-02	C20orf194		
chr11.0031087495	0.90	1.50E-02	DCDC1		
chr15.0031451635	1.32	1.50E-02			
chr13.0079040535	0.93	1.50E-02	RNF219-AS1		
chr20.0055728952	-1.07	1.51E-02			
chr15.0039110124	-1.22	1.51E-02			
chr20.0038724310	1.58	1.51E-02			
chr20.0060933723	1.35	1.51E-02			
chr5.0057845264	0.72	1.51E-02	CTD-2117L12.1		
chr15.0066646204	1.05	1.51E-02	TIPIN		

chr7.0005517660	1.20	1.52E-02	FBXL18, AC092171.4		
chr12.0123942691	1.43	1.52E-02	SNRNP35		
chr14.0100588329	1.29	1.52E-02	EVL		
chr17.0077908058	1.10	1.53E-02	TBC1D16		
chr13.0063769342	0.90	1.53E-02	LINC00376		
chr3.0148729673	1.21	1.53E-02	GYG1		
chr20.0034214499	-0.92	1.53E-02	CPNE1		X
chr11.0094428046	1.13	1.53E-02	RP11-867G2.8		X
chr19.0014089299	-1.25	1.53E-02	RFX1		
chr9.0136600382	0.85	1.53E-02	SARDH		
chr14.0052593986	-0.81	1.54E-02	RP11-1033H12.1		
chr17.0008219019	1.36	1.54E-02	ARHGEF15		
chr12.0122984696	-1.30	1.55E-02	ZCCHC8		
chr11.0132617559	0.82	1.55E-02	OPCML		
chr12.0026672531	1.28	1.56E-02	ITPR2		
chr20.0001813479	-1.42	1.56E-02			
chr3.0004241375	0.85	1.57E-02	SUMF1		
chr15.0041023492	1.01	1.58E-02	RAD51		X
chr12.0049484577	-1.38	1.58E-02	DHH, RP11-386G11.8		
chr11.0020344257	0.84	1.58E-02			
chr9.0066955241	0.83	1.58E-02			
chr15.0065043833	0.81	1.58E-02	RBPMS2		
chr20.0062589386	-1.64	1.58E-02	ZNF512B, CTD-3184A7.4		
chr18.0068629472	0.92	1.58E-02			
chr6.0019413218	0.86	1.58E-02			
chr20.0062259792	1.46	1.59E-02			
chr15.0034108303	0.90	1.59E-02	RYR3		
chr19.0002867898	1.12	1.59E-02	ZNF556		
chr2.0129166371	-1.03	1.60E-02			
chr12.0106148484	0.83	1.60E-02			
chr13.0041752980	-1.01	1.60E-02			
chr20.0062522605	-1.21	1.60E-02	TPD52L2		
chr12.0107831478	-0.72	1.61E-02	BTBD11		
chr20.0061284334	1.30	1.62E-02	SLCO4A1		
chr6.0168843099	1.25	1.62E-02	SMOC2		
chr5.0171756702	-0.83	1.62E-02	SH3PXD2B		
chr20.0023859143	-0.97	1.62E-02	CST5		X
chr6.0166347327	0.86	1.62E-02	PDE10A, LINC00473		
chr12.0031882241	1.81	1.62E-02			
chr5.0058092517	0.83	1.62E-02	RAB3C, RP11-479O16.1		
chr20.0062786731	1.17	1.62E-02	MYT1		

chr6.0099135339	0.89	1.63E-02			
chr22.0044562387	0.98	1.63E-02	PARVB		
chr18.0018706758	-0.92	1.63E-02			
chr19.0015108434	-0.92	1.63E-02	SLC1A6		
chr9.0139395217	0.80	1.63E-02	NOTCH1	X	
chr14.0103649648	0.84	1.64E-02			
chr15.0069854566	1.02	1.64E-02			
chr15.0068872137	1.16	1.64E-02			
chr3.0120314864	0.88	1.64E-02			
chr15.0025955428	0.50	1.64E-02	ATP10A		
chr15.0027786719	0.94	1.64E-02	RP11-100M12.3		
chr3.0055523173	-0.95	1.64E-02	WNT5A		
chr15.0079601574	1.12	1.64E-02			
chr6.0168625864	0.78	1.64E-02			
chr3.0182987901	-0.82	1.65E-02	MCF2L2, B3GNT5		
chr15.0041080358	1.40	1.65E-02	DNAJC17		
chr12.0001858063	0.85	1.65E-02	ADIPOR2		
chr2.0237073838	-1.07	1.65E-02			
chr15.0101866025	-1.19	1.65E-02			
chr3.0012923687	0.81	1.66E-02	RP11-767C1.1		
chr15.0028716221	-0.86	1.66E-02			
chr11.0065358078	1.30	1.66E-02	EHBP1L1		
chr22.0047242843	0.86	1.66E-02	TBC1D22A		
chr20.0044458275	-0.95	1.66E-02	TNNC2		
chr20.0057986154	-0.63	1.67E-02			
chr19.0001367607	0.84	1.67E-02	MUM1		
chr16.0058824222	0.74	1.67E-02	RP11-410D17.2		
chr5.0176295797	0.81	1.67E-02	UNC5A		
chr12.0058003396	1.61	1.67E-02	DTX3		
chr12.0091564554	0.97	1.68E-02	DCN	X	
chr7.0003271465	-0.82	1.68E-02			
chr18.0056046635	-0.66	1.68E-02	NEDD4L	X	
chr20.0062149203	-1.49	1.68E-02			
chr13.0034225558	-1.10	1.68E-02	RP11-141M1.3		
chr17.0035165969	1.07	1.69E-02			
chr22.0025600824	1.10	1.70E-02	CRYBB3		
chr15.0068583382	0.98	1.70E-02	FEM1B		
chr16.0058757515	0.88	1.70E-02	GOT2		
chr16.0004604746	0.77	1.71E-02			
chr22.0019865215	-0.85	1.71E-02	TXNRD2		
chr6.0151404627	-0.84	1.71E-02	MTHFD1L		

chr15.0091398741	1.06	1.71E-02			
chr15.0051203970	1.12	1.72E-02			
chr17.0073633572	-0.75	1.72E-02	RECQL5, SMIM5		
chr12.0029430585	-0.88	1.73E-02	FAR2		
chr13.0099129570	-1.16	1.73E-02	STK24		
chr2.0092014991	1.15	1.73E-02			
chr12.0120832604	1.40	1.73E-02	RP1-166H1.2		
chr6.0027780652	1.16	1.73E-02			
chr14.0024851275	1.46	1.74E-02			X
chr19.0004099560	1.04	1.74E-02	MAP2K2		
chr15.0064816687	-0.75	1.74E-02	ZNF609	X	
chr5.0035853643	-1.07	1.75E-02	IL7R		
chr14.0020835244	-0.88	1.75E-02	TEP1		
chr20.0035325831	0.81	1.76E-02	NDRG3		
chr11.0044343296	0.93	1.76E-02			
chr11.0114042360	1.09	1.76E-02	ZBTB16		
chr15.0079071423	-1.02	1.77E-02			
chr2.0226496814	0.82	1.77E-02	NYAP2		
chr19.0046196188	-1.15	1.77E-02	QPCTL		
chr3.0182830735	-0.82	1.78E-02	MCCC1		
chr22.0032743558	0.81	1.79E-02			
chr15.0082273478	0.84	1.80E-02	RP11-276M12.1		
chr12.0049603111	-0.71	1.81E-02	TUBA1C		
chr2.0033057636	-0.85	1.81E-02	LINC00486		
chr22.0048252957	0.90	1.82E-02			
chr20.0056026012	-0.67	1.83E-02			
chr12.0129310781	-1.27	1.83E-02			
chr17.0042857648	0.91	1.83E-02	ADAM11		
chr12.0008857266	0.81	1.84E-02	RIMKLB		
chr6.0036799126	-0.94	1.84E-02	CPNE5		
chr14.0031513408	1.04	1.84E-02	AP4S1		
chr15.0084324496	0.95	1.84E-02	ADAMTSL3		
chr2.0020377557	-0.98	1.84E-02			
chr15.0067062701	0.73	1.84E-02	SMAD6		
chr17.0017415526	1.11	1.84E-02	PEMT		
chr9.0139001670	-0.83	1.84E-02			
chr9.0094949218	0.97	1.85E-02			
chr14.0099849307	1.10	1.85E-02			X
chr3.0151630654	-0.77	1.85E-02	RP11-454C18.2		
chr15.0063197466	0.93	1.85E-02			
chr15.0064227585	-0.86	1.85E-02	DAPK2, RP11-111E14.1		

chr16.0025617907	0.79	1.85E-02			
chr18.0068364888	1.23	1.85E-02			
chr5.0178715673	-1.21	1.85E-02	ADAMTS2		
chr11.0061341207	0.88	1.86E-02	SYT7		
chr18.0046834838	-0.90	1.86E-02	DYM		
chr13.0051396659	1.42	1.86E-02	DLEU7, DLEU7-AS1		
chr14.0024778911	0.90	1.86E-02	CIDEB, LTB4R2		
chr10.0025246705	1.02	1.86E-02			
chr3.0108046904	-0.82	1.87E-02	HHLA2		
chr11.0123445488	0.80	1.87E-02	GRAMD1B		
chr12.0054131365	0.90	1.88E-02			
chr2.0005433241	-0.95	1.88E-02			
chr8.0126981483	-1.01	1.88E-02			
chr22.0033886653	0.85	1.88E-02	LARGE1	X	X
chr20.0058035543	0.89	1.88E-02			
chr18.0045862942	-1.36	1.88E-02			
chr15.0071754544	-0.74	1.89E-02	THSD4		
chr5.0007824030	1.10	1.89E-02	ADCY2		
chr9.0038621990	1.68	1.89E-02	FAM201A		
chr13.0042614083	-0.82	1.89E-02			
chr12.0000662536	1.56	1.89E-02			
chr13.0115001386	1.06	1.90E-02			
chr18.0072175316	0.89	1.90E-02	CNDP2		
chr22.0039967081	0.85	1.90E-02	CACNA1I		
chr2.0099422979	1.14	1.92E-02	KIAA1211L		
chr11.0011613810	0.90	1.94E-02	GALNT18		
chr3.0037333531	0.95	1.95E-02			
chr12.0092402155	0.95	1.96E-02	C12orf79		
chr12.0001087688	0.73	1.96E-02	RAD52, RP11-359B12.2		
chr19.0052992415	0.72	1.97E-02	ZNF578		
chr12.0055783991	1.29	1.97E-02			
chr2.0098487920	0.84	1.97E-02	TMEM131		
chr2.0116280845	-0.88	1.98E-02	DPP10		
chr16.0000583839	1.15	1.98E-02	CAPN15		
chr18.0047250077	-1.10	1.98E-02			
chr14.0105048321	1.18	1.98E-02	C14orf180		
chr11.0017693024	0.97	1.98E-02			
chr11.0001037098	-1.11	2.00E-02			
chr6.0046368470	0.79	2.01E-02	RCAN2		
chr15.0088721801	0.99	2.01E-02			
chr13.0041995963	-0.74	2.01E-02			X

chr12.0023350005	0.83	2.01E-02	RP11-153K16.2		
chr16.0022205553	0.85	2.02E-02			
chr11.0071150631	-0.84	2.03E-02	DHCR7		
chr15.0084912101	-1.35	2.05E-02	GOLGA6L4		
chr16.0078140608	-0.83	2.05E-02	WWOX		
chr9.0075217586	-1.13	2.05E-02	TMC1		
chr4.0112728255	0.66	2.05E-02	RP11-269F21.1, RP11-269F21.2		
chr10.0108520582	-0.76	2.06E-02	SORCS1		
chr12.0110438723	1.19	2.06E-02	ANKRD13A		
chr14.0096552963	-0.90	2.06E-02	C14orf132		
chr12.0131418766	-0.94	2.07E-02			
chr11.0031121358	-0.76	2.07E-02	DCDC1		
chr19.0033896553	-1.31	2.07E-02	PEPD		
chr18.0057470391	-0.82	2.07E-02			
chr13.0021286449	-1.33	2.07E-02	IL17D		
chr5.0001640848	-1.11	2.07E-02			
chr15.0093906869	0.84	2.08E-02	RP11-266O8.1		
chr11.0003440420	-0.80	2.08E-02	FAM86GP		
chr14.0100427761	-0.93	2.08E-02			
chr3.0027929607	0.83	2.08E-02	AC098973.2		
chr17.0034501886	1.43	2.09E-02	CTB-91J4.1, TBC1D3B		
chr18.0045057574	-1.46	2.10E-02	CTD-2130O13.1, RP11-157P23.2		
chr22.0039006789	0.79	2.10E-02	FAM227A		
chr5.0039219698	1.37	2.10E-02	FYB		
chr20.0024958641	-0.50	2.10E-02	APMAP		
chr9.0092333879	0.89	2.10E-02	RP11-316P17.2		
chr13.0089273198	0.87	2.10E-02			
chr14.0024785859	-1.21	2.10E-02	LTB4R	X	
chr12.0088429374	0.69	2.10E-02	C12orf29		
chr11.0134746717	0.97	2.10E-02			
chr6.0075912320	1.13	2.10E-02	COL12A1		
chr20.0049342667	0.93	2.10E-02			
chr10.0065475234	-0.71	2.11E-02	RP11-170M17.1		
chr12.0024898838	0.93	2.12E-02	RP11-625L16.1		
chr14.0102059428	0.88	2.13E-02			
chr3.0007060708	0.79	2.14E-02	GRM7		
chr8.0133070210	1.02	2.15E-02	OC90		X
chr13.0094140396	0.79	2.15E-02	GPC6		
chr17.0059750714	-0.82	2.15E-02			
chr18.0045686939	-0.69	2.15E-02	ZBTB7C		
chr18.0071611180	-0.99	2.15E-02	RP11-25L3.3		

chr20.0015588271	0.85	2.16E-02	MACROD2		
chr6.0161063597	-1.62	2.16E-02	LPA		
chr20.0042887008	0.97	2.16E-02	GDAP1L1		
chr4.0112764777	0.65	2.17E-02	RP11-269F21.3		
chr16.0079166247	0.85	2.17E-02	RP11-679B19.2		
chr16.0079250798	0.76	2.17E-02	RP11-679B19.2		
chr3.0141163289	-0.83	2.17E-02	ZBTB38		
chr12.0115161241	-0.82	2.17E-02	RP4-601P9.2		
chr17.0062777136	-1.17	2.18E-02	hsa-mir-6080, PLEKHM1P		
chr16.0089778878	-1.08	2.18E-02	VPS9D1, VPS9D1-AS1		
chr12.0033048797	2.07	2.18E-02	PKP2	X	
chr15.0066311980	1.36	2.18E-02	MEGF11		X
chr11.0132475539	0.85	2.18E-02	OPCML		
chr22.0050508487	-0.80	2.18E-02	MLC1		
chr11.0066315845	1.15	2.18E-02	ACTN3	X	
chr13.0096687076	-0.81	2.19E-02	UGGT2		
chr12.0057856280	1.43	2.19E-02	GLI1		
chr18.0038321088	0.86	2.20E-02			
chr5.0019575004	-0.75	2.20E-02	CDH18		
chr11.0120447543	0.78	2.20E-02	GRIK4		
chr13.0026538260	1.26	2.21E-02	ATP8A2	X	X
chr22.0024547472	0.78	2.21E-02	CABIN1		
chr20.0001821959	-1.35	2.21E-02			
chr14.0023070923	-1.29	2.21E-02	ABHD4		
chr6.0164042394	-0.87	2.22E-02			
chr16.0070207297	-1.02	2.22E-02	RP11-296I10.3, CLEC18C		
chr12.0113690924	-0.84	2.22E-02	TPCN1		
chr10.0068355194	-0.77	2.23E-02	CTNNA3		
chr22.0040358235	0.92	2.23E-02	GRAP2, RP3-370M22.8		
chr2.0003882321	0.90	2.24E-02			
chr19.0006913486	1.03	2.24E-02	EMR1		
chr20.0061767784	0.95	2.27E-02			
chr22.0037302861	0.99	2.27E-02			
chr17.0019035897	-0.95	2.27E-02	GRAPL		
chr18.0074151887	-0.54	2.27E-02	ZNF516		
chr11.0033420794	-0.76	2.27E-02			
chr17.0040439892	1.08	2.27E-02	STAT5A		
chr12.0113684022	1.97	2.27E-02			
chr20.0062257281	-0.72	2.27E-02	GMEB2		
chr6.0151652742	0.85	2.27E-02	AKAP12		
chr12.0085672242	0.89	2.27E-02			

chr2.0233983675	1.05	2.27E-02	INPP5D		
chr12.0005562121	0.79	2.29E-02	NTF3		
chr12.0125131378	1.10	2.30E-02			
chr22.0042606263	-0.80	2.31E-02	TCF20		
chr12.0131691177	-1.19	2.31E-02	RP11-638F5.1		
chr3.0018656789	0.75	2.32E-02	AC144521.1		
chr6.0003438536	0.78	2.32E-02	SLC22A23		
chr12.0110224997	1.05	2.34E-02	TRPV4		
chr16.0057334568	-1.47	2.34E-02			
chr12.0009931166	0.78	2.35E-02			
chr12.0027574674	0.92	2.35E-02	ARNTL2, RP11-165P7.1		
chr22.0037997972	-1.02	2.35E-02			
chr15.0085245022	-0.81	2.35E-02	SEC11A		
chr3.0169444964	-0.83	2.35E-02			
chr2.0222301426	0.76	2.36E-02	EPHA4		
chr10.0071267921	-0.84	2.36E-02			
chr10.0117820694	0.84	2.36E-02	GFRA1		
chr16.0011595555	-1.04	2.37E-02	CTD-3088G3.8		
chr12.0024994558	1.02	2.37E-02	BCAT1		
chr16.0084857412	-1.06	2.37E-02	CRISPLD2		
chr6.0013510456	0.86	2.37E-02			
chr12.0115255939	-1.45	2.37E-02			
chr13.0053624408	0.80	2.37E-02	OLFM4		
chr2.0211575755	0.94	2.38E-02			
chr8.0145046909	1.10	2.39E-02	PLEC		
chr6.0053524907	0.84	2.39E-02	KLHL31		
chr12.0025937825	1.36	2.39E-02	RP11-443N24.2		
chr11.0125773076	-1.02	2.39E-02	PUS3		
chr18.0010315725	0.88	2.40E-02			
chr14.0024827522	-0.87	2.40E-02			
chr15.0080588199	-0.81	2.40E-02	LINC00927		
chr2.0086023752	-1.17	2.40E-02			
chr2.0006950261	1.00	2.40E-02			
chr2.0103747925	-0.71	2.40E-02	AC073987.1		
chr14.0097571434	0.93	2.40E-02			
chr19.0000608811	0.97	2.40E-02	HCN2		
chr15.0079233994	0.79	2.41E-02	CTSH		
chr18.0072250823	1.05	2.41E-02	CNDP1		
chr3.0119813692	1.83	2.41E-02			
chr19.0000751159	0.86	2.42E-02	MISP		
chr16.0085744228	1.23	2.43E-02	C16orf74		

chr6.0116464670	-1.06	2.44E-02	NT5DC1, COL10A1		
chr15.0065563447	1.20	2.44E-02	PARP16		
chr15.0052550022	1.03	2.44E-02	MYO5C		
chr6.0022021274	0.83	2.45E-02	CASC15		
chr16.0082451812	0.86	2.45E-02			
chr11.0134548961	0.98	2.47E-02			
chr2.0227661381	0.88	2.48E-02	IRS1		
chr12.0057634891	-1.27	2.48E-02			
chr16.0083174768	0.72	2.48E-02	CDH13		
chr19.0038215633	-0.69	2.48E-02			
chr17.0015380552	0.78	2.48E-02	TVP23C-CDRT4, TVP23C, CDRT4		
chr12.0018568899	-0.79	2.48E-02	PIK3C2G		
chr11.0062357965	-0.84	2.49E-02	MIR3654, TUT1		
chr20.0007521120	0.81	2.49E-02			
chr3.0151160316	-0.79	2.49E-02	IGSF10		
chr20.0037174617	0.91	2.49E-02	RALGAPB		
chr6.0064193431	-0.99	2.49E-02			
chr2.0039419989	-0.78	2.49E-02	CDKL4		
chr16.0005263031	-0.91	2.49E-02			
chr20.0031210733	1.36	2.50E-02			
chr15.0101536131	0.82	2.50E-02	LRRK1		
chr5.0179114073	0.76	2.50E-02	CANX, HMGB3P22		
chr11.0134214876	0.96	2.50E-02	GLB1L2		
chr3.0151203613	-0.77	2.51E-02			
chr9.0095906540	1.14	2.51E-02			
chr2.0217964246	0.74	2.51E-02			X
chr9.0112810447	0.92	2.51E-02	PALM2-AKAP2, AKAP2		
chr12.0049409447	0.89	2.51E-02	RP11-386G11.5, PRKAG1		
chr17.0012769886	0.92	2.51E-02	ARHGAP44		
chr3.0046503548	-1.14	2.51E-02	LTF		
chr7.0100812136	0.94	2.51E-02			
chr6.0016993940	0.81	2.52E-02			
chr11.0061310165	0.86	2.52E-02	SYT7		
chr20.0007889631	0.75	2.52E-02	HAO1		
chr17.0012479544	0.81	2.53E-02	LINC00670		
chr10.0049211544	-1.00	2.53E-02			
chr4.0137501021	0.71	2.53E-02			
chr13.0026590625	0.87	2.53E-02			
chr3.0025114359	0.78	2.53E-02	AC133680.1		
chr11.0043605047	1.24	2.54E-02	HSD17B12		
chr14.0092901413	0.92	2.54E-02	SLC24A4		

chr6.0032038784	-1.12	2.55E-02	TNXB		
chr3.0052345311	1.17	2.55E-02			
chr18.0010485679	0.82	2.55E-02	APCDD1		
chr16.0068174112	0.73	2.55E-02	NFATC3		
chr5.0172106425	-0.80	2.56E-02	NEURL1B		
chr8.0133928368	-0.69	2.56E-02	TG		
chr19.0048669578	0.70	2.57E-02	LIG1		
chr9.0127203900	0.76	2.57E-02			
chr12.0116797924	1.11	2.57E-02			
chr18.0057193836	-1.06	2.57E-02	CCBE1		
chr12.0131863915	-0.80	2.57E-02			
chr14.0080484300	1.32	2.57E-02			
chr17.0015394553	1.18	2.58E-02			
chr10.0098457342	0.66	2.58E-02	PIK3AP1		
chr10.0028726670	0.83	2.58E-02	RP11-351M16.3		
chr3.0055518374	0.96	2.59E-02	WNT5A	X	
chr14.0073686279	-0.82	2.59E-02	PSEN1		
chr20.0059215410	0.90	2.60E-02			
chr19.0039622344	1.24	2.61E-02	PAK4		
chr10.0135088481	-0.99	2.61E-02	ADAM8		
chr18.0020959985	-1.40	2.61E-02	TMEM241		
chr16.0029187478	0.79	2.62E-02	RP11-426C22.5		
chr12.0130600247	0.95	2.62E-02			
chr9.0130395772	0.86	2.62E-02	STXBP1		
chr13.0099295245	-0.79	2.63E-02			
chr16.0058789143	0.98	2.63E-02	RP11-410D17.2		
chr20.0051420595	-0.85	2.64E-02			
chr18.0013140322	-0.84	2.64E-02	RP11-807E13.3		
chr17.0007260381	-1.16	2.64E-02	TMEM95		
chr8.0143193665	-0.90	2.64E-02			
chr20.0003661537	-0.70	2.64E-02	ADAM33		
chr15.0088583056	0.93	2.64E-02	NTRK3		
chr11.0074247056	-1.13	2.66E-02	POLD3		
chr12.0062584454	2.13	2.67E-02	FAM19A2		
chr15.0068126938	0.92	2.67E-02	RP11-34F13.3, RP11-34F13.2		
chr18.0054716316	-0.74	2.67E-02			
chr12.0005069874	0.77	2.67E-02			
chr2.0052278383	-0.89	2.67E-02	AC007682.1		
chr12.0004810242	1.06	2.68E-02	RP11-234B24.6, RP11-234B24.2		
chr12.0003126663	0.82	2.68E-02	TEAD4	X	
chr9.0140239835	0.71	2.68E-02	EXD3		

chr20.0017899412	0.79	2.69E-02			
chr9.0079448403	0.77	2.70E-02	PRUNE2		
chr5.0105341037	-0.80	2.70E-02			
chr15.0051604337	0.66	2.72E-02	CYP19A1		
chr3.0020088712	-1.20	2.74E-02	KAT2B		
chr3.0174382932	0.84	2.74E-02	NAALADL2		
chr15.0069794562	0.86	2.75E-02	RP11-279F6.1		
chr15.0050280046	-1.14	2.75E-02	ATP8B4		
chr9.0140352405	-0.84	2.75E-02	NSMF		
chr22.0016645749	-1.03	2.75E-02	ZNF72P		
chr15.0070105816	0.92	2.75E-02			
chr12.0099615042	0.85	2.75E-02	ANKS1B		
chr22.0048641448	-0.84	2.78E-02			X
chr22.0038507208	0.82	2.79E-02			
chr3.0046490317	-0.88	2.79E-02			
chr21.0046301789	-0.77	2.79E-02			
chr19.0007069967	0.95	2.79E-02	ZNF557		
chr12.0130962583	0.84	2.79E-02	RIMBP2		
chr2.0240905174	0.91	2.80E-02	NDUFA10		
chr14.0064598744	0.76	2.80E-02	SYNE2, ESR2		
chr13.0103185856	-0.91	2.80E-02			
chr8.0005458906	0.75	2.80E-02			
chr20.0061428843	-1.18	2.80E-02	MRGBP		
chr13.0028550394	-1.35	2.80E-02			
chr18.0044478678	0.80	2.80E-02	PIAS2		
chr14.0101841897	0.85	2.80E-02			
chr20.0011633534	0.86	2.83E-02			
chr7.0129996892	0.96	2.83E-02	CPA5		
chr6.0015896174	0.91	2.83E-02			
chr18.0019067987	-0.72	2.83E-02	GREB1L		
chr12.0125017105	0.91	2.83E-02	NCOR2		
chr17.0055520767	0.99	2.84E-02	MSI2		
chr14.0103740089	1.33	2.84E-02			
chr12.0101787956	0.89	2.84E-02	ARL1		
chr2.0216879665	0.75	2.86E-02	MREG, PECR		
chr6.0146505684	0.81	2.86E-02	GRM1		
chr8.0010191232	0.92	2.86E-02	MSRA		
chr18.0008264490	-0.88	2.86E-02	PTPRM		X
chr15.0069952346	0.84	2.86E-02	RP11-279F6.3		
chr6.0064254064	-1.13	2.87E-02	PTP4A1		X
chr20.0062134308	-1.16	2.87E-02	RP4-697K14.3		

chr20.0045845539	0.97	2.87E-02	ZMYND8		
chr6.0090109650	0.80	2.87E-02	RRAGD		
chr14.0102047819	0.81	2.88E-02			
chr20.0051361280	0.70	2.88E-02			
chr13.0114802848	-1.14	2.89E-02	RASA3		
chr14.0039262070	0.95	2.90E-02	LINC00639		
chr3.0060919598	-0.86	2.90E-02	FHIT		
chr14.0100142498	1.27	2.90E-02	HHIPL1		X
chr13.0057743067	1.49	2.90E-02	PRR20E		
chr20.0019192507	1.60	2.91E-02			
chr12.0105820659	0.92	2.92E-02			
chr15.0041696379	-0.89	2.92E-02			
chr13.0041773854	0.91	2.92E-02			X
chr5.0011248947	0.82	2.92E-02	CTNND2		
chr5.0109607702	0.70	2.93E-02			
chr3.0153560971	0.81	2.95E-02	RP11-23D24.2		
chr6.0064215203	-0.91	2.95E-02			
chr18.0025139835	0.87	2.95E-02	RP11-739N10.1		
chr22.0027286206	0.84	2.95E-02			
chr3.0017562733	0.85	2.95E-02	TBC1D5		
chr11.0069688120	-1.00	2.95E-02			
chr19.0043892591	-0.95	2.96E-02			X
chr9.0006183267	0.77	2.96E-02			
chr6.0026856434	-1.45	2.97E-02	GUSBP2		
chr20.0047137619	0.92	2.98E-02			
chr20.0006101929	-0.89	2.98E-02	FERMT1		
chr14.0101661156	0.82	2.98E-02			
chr2.0024165918	-0.76	2.98E-02	UBXN2A		
chr4.0153641626	0.88	2.98E-02			X
chr3.0068791660	0.99	2.98E-02	FAM19A4		
chr11.0066364146	0.74	2.98E-02	CCS		
chr5.0053421823	-0.75	2.99E-02	ARL15		
chr5.0141659848	-1.25	2.99E-02			
chr8.0031067834	-0.90	2.99E-02			
chr14.0019604345	-1.62	3.00E-02			
chr12.0066049238	1.02	3.00E-02	RP11-221N13.4		
chr17.0003462368	1.01	3.00E-02			
chr12.0001400446	0.95	3.00E-02	ERC1		
chr3.0194674177	1.15	3.00E-02			
chr12.0053678199	0.86	3.00E-02	ESPL1		
chr15.0096647029	-1.11	3.00E-02			

chr16.0069662747	-0.74	3.01E-02	NFAT5		
chr12.0111902693	0.87	3.01E-02	ATXN2		
chr14.0089721594	-1.01	3.01E-02	FOXN3		
chr20.0060645662	-0.51	3.01E-02			
chr9.0137475754	0.76	3.02E-02			
chr9.0030518167	0.79	3.03E-02			
chr3.0123550508	0.84	3.04E-02	MYLK	X	
chr5.0022752463	0.79	3.04E-02	CDH12		
chr17.0046221813	0.89	3.04E-02	SKAP1		
chr11.0003144685	1.45	3.04E-02	OSBPL5		
chr7.0147581299	-0.83	3.04E-02	CNTNAP2		
chr12.0003241735	1.04	3.05E-02	TSPAN9		
chr10.0133276830	-1.03	3.05E-02			
chr2.0120041552	1.08	3.05E-02			
chr6.0102097023	0.79	3.05E-02	GRIK2		
chr20.0025725007	-0.78	3.05E-02			
chr17.0043356139	-0.97	3.05E-02	MAP3K14		
chr9.0139651444	0.79	3.05E-02	LCN8		
chr8.0008003698	0.78	3.05E-02			
chr16.0083606272	0.86	3.06E-02			
chr15.0039802950	1.00	3.06E-02			X
chr9.0117623760	0.87	3.06E-02			
chr19.0035611941	0.88	3.06E-02	FXYD3		
chr14.0104178670	1.04	3.06E-02	XRCC3, AL049840.1		
chr14.0077398971	1.00	3.07E-02			
chr14.0078123724	0.97	3.07E-02			
chr15.0057403882	-0.99	3.08E-02	TCF12	X	
chr15.0063368030	1.15	3.08E-02			
chr18.0056146980	0.84	3.11E-02			
chr8.0008300606	-0.90	3.11E-02			
chr15.0044284871	0.86	3.11E-02	FRMD5		
chr11.0071049344	0.76	3.12E-02			
chr3.0047116517	-0.76	3.13E-02	SETD2		
chr11.0111316435	-0.86	3.13E-02	POU2AF1		
chr17.0006001954	1.04	3.13E-02	WSCD1		
chr16.0087698990	-0.90	3.14E-02	JPH3		
chr13.0051846552	0.77	3.14E-02	FAM124A		
chr18.0056046953	-0.80	3.14E-02	NEDD4L	X	
chr17.0020909292	-1.05	3.14E-02	USP22		
chr20.0031129482	1.35	3.14E-02	C20orf112		
chr11.0118663188	-1.12	3.14E-02			

chr15.0073631717	0.97	3.15E-02	HCN4, RP11-272D12.1	X	
chr5.0149566637	-1.06	3.15E-02			
chr3.0014041701	0.73	3.16E-02	TPRXL		
chr12.0050448047	-0.77	3.17E-02			
chr11.0020039824	-0.75	3.17E-02	NAV2		
chr19.0013136364	0.97	3.17E-02	NFIX		
chr15.0091398642	1.21	3.17E-02			
chr6.0057602917	0.99	3.18E-02			
chr6.0034463322	-0.78	3.18E-02	PACSIN1		
chr14.0086228692	0.97	3.18E-02			X
chr16.0058790064	0.92	3.18E-02			
chr17.0016182623	0.71	3.19E-02	PIGL		
chr12.0123753032	1.83	3.19E-02	CDK2AP1		
chr13.0100145409	0.94	3.19E-02			
chr20.0060061483	0.92	3.19E-02	CDH4		
chr16.0027852176	0.73	3.19E-02	GSG1L		
chr9.0134184484	0.75	3.19E-02	PPAPDC3		
chr14.0100291764	0.84	3.19E-02	EML1		
chr17.0035425610	-1.12	3.19E-02			
chr12.0123135330	0.99	3.19E-02	HCAR1		
chr18.0012466006	0.79	3.19E-02	SPIRE1		
chr9.0134177501	1.14	3.19E-02			
chr13.0037125617	0.87	3.20E-02			
chr5.0001359318	0.88	3.20E-02			
chr18.0034431276	-0.97	3.20E-02	KIAA1328		X
chr10.0080956875	0.98	3.20E-02	ZMIZ1		
chr18.0003580666	0.77	3.20E-02	DLGAP1, RP11-710M11.1		
chr15.0101700896	0.91	3.21E-02			
chr18.0029303612	-0.65	3.21E-02			
chr22.0040127961	0.90	3.21E-02			X
chr16.0010906798	1.14	3.21E-02	TVP23A		
chr13.0114071457	-1.12	3.21E-02			
chr3.0043677568	0.73	3.21E-02	ANO10		
chr14.0071251384	0.80	3.21E-02	MAP3K9		
chr11.0132717823	0.93	3.21E-02			
chr17.0043631042	-0.87	3.21E-02	RP11-798G7.6, RP11-798G7.7		X
chr12.0083248478	0.87	3.21E-02	TMTC2		
chr14.0023201418	0.88	3.21E-02	CTD-2555K7.2		
chr12.0006650910	1.16	3.21E-02	IFFO1		
chr3.0084228558	0.84	3.21E-02			
chr8.0067351838	0.96	3.22E-02	ADHFE1		

chr18.0056064648	0.82	3.22E-02			X
chr18.0040084697	0.88	3.22E-02	LINC00907		
chr12.0001414241	1.01	3.22E-02			
chr13.0036104053	-0.83	3.24E-02	NBEA		
chr20.0033566149	0.86	3.24E-02	MYH7B		
chr7.0143885174	0.84	3.24E-02	ARHGEF35		
chr15.0078122914	-1.02	3.25E-02			X
chr20.0059336133	0.88	3.25E-02			
chr20.0055066923	0.80	3.25E-02	RTFDC1, GCNT7		
chr16.0029296720	-1.00	3.25E-02	SNX29P2		
chr3.0052074225	1.20	3.25E-02			
chr12.0114887448	0.71	3.26E-02			
chr13.0042769050	-0.86	3.26E-02	DGKH		
chr17.0020940382	-1.05	3.26E-02			
chr14.0077523673	-0.74	3.26E-02	RP11-7F17.5, RP11-7F17.4		
chr17.0027056417	0.84	3.26E-02	NEK8		
chr12.0067087331	0.82	3.27E-02	GRIP1		
chr8.0126703953	1.02	3.27E-02			
chr2.0102623275	-0.96	3.27E-02	IL1R2		
chr5.0010571406	0.73	3.27E-02	ANKRD33B		
chr20.0045457978	-0.99	3.27E-02			X
chr12.0001312523	0.94	3.27E-02			
chr11.0001245363	0.75	3.28E-02	MUC5B		
chr5.0000534067	0.96	3.28E-02			
chr20.0006759498	-0.98	3.28E-02	BMP2	X	
chr8.0027132793	0.87	3.28E-02			
chr2.0037555152	0.80	3.28E-02			
chr18.0038158241	1.28	3.28E-02			
chr22.0018947523	0.87	3.29E-02			
chr9.0085110033	0.78	3.30E-02	RP11-15B24.5		
chr17.0036059462	0.99	3.30E-02	HNF1B		
chr12.0054366343	1.07	3.30E-02	HOTAIR		
chr2.0110781416	0.91	3.30E-02			
chr15.0036298829	0.47	3.30E-02	RP11-184D12.1		X
chr11.0000732316	-0.86	3.30E-02			
chr14.0034000223	-0.78	3.32E-02	NPAS3		
chr12.0050942855	-0.64	3.32E-02	DIP2B		
chr13.0026508624	0.80	3.33E-02			
chr19.0002032975	0.96	3.34E-02	BTBD2		
chr2.0010830783	0.97	3.34E-02	RN7SL832P		
chr17.0016744139	0.81	3.34E-02	KRT17P1		

chr10.0133843269	0.79	3.34E-02			
chr14.0080458717	0.78	3.34E-02			
chr19.0056974990	-0.66	3.34E-02	ZNF667		
chr11.0113209030	0.91	3.34E-02	TTC12		
chr2.0163392567	0.86	3.34E-02	KCNH7		
chr18.0049055189	0.85	3.35E-02	RP11-267C16.1		
chr19.0005040201	0.71	3.36E-02	KDM4B		
chr5.0138906155	0.83	3.36E-02	UBE2D2		
chr8.0006631730	0.88	3.36E-02			
chr20.0060852455	-1.48	3.36E-02	OSBPL2		
chr3.0142010930	0.73	3.37E-02			
chr17.0040699809	-0.94	3.37E-02	RP11-400F19.8, HSD17B1P1		
chr20.0035516795	-1.04	3.37E-02	TLDC2		
chr17.0079561157	-0.73	3.37E-02	NPLOC4		
chr13.0023711332	1.05	3.37E-02			
chr13.0027300958	0.80	3.38E-02			
chr11.0047470312	1.21	3.38E-02	RAPSN		
chr18.0057872243	-0.61	3.39E-02			
chr18.0041832689	-0.67	3.39E-02			
chr6.0035778755	-0.90	3.39E-02	LHFPL5		
chr20.0036839379	1.39	3.39E-02	KIAA1755		
chr5.0000419577	-0.92	3.39E-02	AHRR		
chr6.0041577098	0.76	3.40E-02			
chr3.0070526101	-0.95	3.40E-02			X
chr22.0020278589	-0.94	3.40E-02			
chr12.0130317295	1.01	3.43E-02	TMEM132D		
chr8.0144390809	-0.68	3.43E-02	TOP1MT		
chr17.0034580276	1.58	3.43E-02			
chr15.0092929384	0.82	3.43E-02			
chr12.0125084212	0.82	3.44E-02			
chr2.0101618347	-1.50	3.44E-02	AC016738.4, RPL31		
chr22.0016882915	0.92	3.45E-02			
chr13.0053864412	0.94	3.47E-02			
chr11.0031377518	0.84	3.48E-02			
chr13.0034217766	1.31	3.49E-02	RP11-141M1.3		
chr20.0036816189	1.35	3.49E-02			
chr3.0073253115	0.75	3.49E-02			
chr19.0004633885	0.91	3.49E-02			
chr14.0055310671	0.89	3.49E-02	GCH1		
chr6.0027775525	0.90	3.49E-02	HIST1H2BL		
chr16.0024360601	-0.88	3.50E-02	CACNG3		X

chr12.0101992093	0.69	3.50E-02	MYBPC1	X	
chr16.0073089170	0.95	3.50E-02	ZFHX3	X	
chr8.0032435807	0.93	3.50E-02	NRG1	X	
chr15.0025916524	1.19	3.50E-02			
chr11.0007563239	-1.17	3.50E-02	PPFIBP2		
chr12.0015366373	-0.93	3.50E-02	RERG		
chr12.0085672520	0.91	3.51E-02			
chr2.0187510417	-0.74	3.51E-02	ITGAV, AC017101.10		
chr18.0034981424	0.86	3.52E-02	CELF4		
chr13.0025261023	-0.88	3.52E-02	ATP12A		
chr13.0087523944	-0.73	3.52E-02			
chr3.0085690798	0.79	3.52E-02	CADM2		
chr13.0094965396	-1.06	3.52E-02			
chr14.0019584879	-1.09	3.52E-02	POTEG		
chr3.0182933209	0.94	3.52E-02	MCF2L2		
chr20.0003253593	-0.80	3.52E-02	C20orf194		
chr17.0043116186	1.10	3.52E-02	DCAKD		
chr9.0134177118	0.74	3.52E-02			
chr18.0075254748	0.81	3.52E-02	RP11-176N18.2		
chr12.0086658897	-0.69	3.52E-02	MGAT4C		
chr3.0124764584	-0.82	3.52E-02	HEG1	X	
chr3.0063474931	1.20	3.52E-02	SYNPR, SYNPR-AS1		
chr12.0083294324	-0.89	3.52E-02			
chr20.0009570410	-0.88	3.53E-02	PAK7		X
chr14.0074684538	-1.50	3.53E-02			
chr2.0037891039	-0.68	3.53E-02	CDC42EP3		
chr18.0009911881	-0.55	3.55E-02			
chr6.0043545539	0.78	3.55E-02	POLH		
chr11.0009890439	-0.75	3.55E-02	SBF2, RP11-1H15.2		X
chr14.0101293014	0.91	3.55E-02	MEG3		
chr20.0041200406	0.99	3.56E-02	PTPRT		
chr17.0001372384	0.91	3.57E-02	MYO1C		
chr12.0056102222	-1.35	3.58E-02	ITGA7	X	
chr11.0078611395	0.83	3.58E-02	TENM4		
chr16.0083085241	0.95	3.58E-02			
chr12.0086907892	-1.60	3.58E-02			
chr9.0133773841	0.96	3.58E-02			
chr15.0074468341	0.96	3.59E-02	RP11-665J16.1, ISLR		
chr20.0057899754	-1.54	3.59E-02	EDN3	X	
chr15.0068926667	0.83	3.60E-02	CORO2B		
chr14.0068040530	0.89	3.60E-02	PLEKHH1		

chr20.0062795688	0.86	3.60E-02	MYT1		
chr13.0099138076	-1.05	3.60E-02	STK24		
chr3.0157697371	0.77	3.60E-02			
chr11.0079647800	0.86	3.61E-02			
chr3.0069652360	-0.81	3.61E-02			
chr15.0033687699	1.03	3.61E-02	RYR3		
chr18.0061925980	-0.98	3.62E-02	RP11-146N18.1, RP11-909B2.1		
chr11.0133526342	-1.06	3.62E-02			
chr14.0076013999	-0.84	3.62E-02			
chr18.0010479529	-0.99	3.62E-02			
chr12.0054425446	-0.89	3.63E-02	RP11-834C11.12, HOXC4, RP11-834C11.14		
chr20.0030700421	-0.80	3.63E-02	TM9SF4		
chr6.0064895861	-0.73	3.63E-02	EYS		
chr2.0200709007	0.82	3.64E-02	FTCDNL1		X
chr2.0029239643	1.01	3.64E-02	FAM179A		
chr15.0074468240	0.97	3.64E-02			
chr17.0065820540	0.98	3.66E-02			
chr13.0038159653	0.85	3.67E-02	POSTN		
chr7.0157379472	0.65	3.67E-02	PTPRN2		
chr20.0059329248	1.03	3.68E-02			
chr16.0007362102	0.84	3.69E-02	RBFOX1		
chr3.0185230852	-0.92	3.69E-02	LIPH		
chr18.0020846541	0.83	3.69E-02			
chr20.0061962018	-0.56	3.70E-02	COL20A1		X
chr3.0005227732	1.01	3.71E-02	AC026202.3		
chr18.0074881019	-0.89	3.71E-02			
chr2.0228610529	0.75	3.72E-02			
chr12.0064763138	-0.80	3.72E-02	RPS11P6, C12orf56		
chr18.0057121773	-0.68	3.72E-02			
chr12.0110906540	1.13	3.72E-02	GPN3, FAM216A		
chr17.0016607041	0.71	3.72E-02	CCDC144A, RP11-219A15.1		
chr6.0116421257	1.40	3.72E-02			
chr9.0138695786	0.82	3.73E-02			
chr16.0021610035	1.52	3.74E-02	METTL9		
chr15.0101207241	0.93	3.74E-02			
chr13.0114004596	0.85	3.74E-02	GRTP1		
chr3.0107987605	0.75	3.75E-02	GPR156		
chr3.0119990864	-1.01	3.75E-02	GPR156		
chr14.0065692418	-0.98	3.76E-02			
chr6.0006002032	0.78	3.76E-02	NRN1		
chr16.0072961680	1.09	3.76E-02			

chr15.0061098639	-0.79	3.76E-02	RORA		
chr12.0005616394	0.83	3.76E-02			X
chr13.0046244319	-1.04	3.77E-02			
chr14.0030107968	-0.78	3.77E-02	PRKD1		
chr12.0013475404	0.91	3.77E-02			
chr5.0097421150	-0.67	3.77E-02			
chr17.0080427574	0.71	3.78E-02	NARF		
chr5.0002753335	-0.79	3.78E-02	C5orf38		
chr20.0042103804	-0.80	3.79E-02			
chr14.0035050452	0.72	3.79E-02	SNX6		
chr17.0066410246	-1.08	3.80E-02	ARSG, RP11-120M18.2		
chr15.0052013314	0.87	3.80E-02			
chr22.0021387675	-0.78	3.80E-02	AC002472.11		
chr17.0001104049	0.79	3.82E-02	ABR		
chr20.0046916138	-1.28	3.82E-02			
chr9.0090919796	0.69	3.82E-02			
chr6.0046597757	-1.13	3.82E-02	CYP39A1		
chr12.0051237500	0.84	3.82E-02	TMPRSS12		
chr13.0107406162	0.78	3.82E-02			
chr2.0032589400	0.72	3.82E-02	BIRC6		
chr6.0123786070	0.67	3.83E-02	TRDN, RP11-532N4.2	X	
chr20.0030437761	-1.43	3.83E-02	DUSP15, EDEM2		
chr13.0037521933	0.79	3.83E-02			
chr20.0015164708	0.81	3.83E-02			
chr20.0033803924	0.80	3.83E-02			
chr6.0108307585	-0.94	3.83E-02			
chr16.0058802517	0.90	3.85E-02			
chr2.0206422383	0.77	3.85E-02	PARD3B		
chr19.0019550548	-0.74	3.85E-02	GATAD2A		
chr19.0034395854	-1.18	3.86E-02			
chr14.0068030779	0.81	3.86E-02			
chr20.0019952933	0.78	3.86E-02	RIN2		
chr18.0069465111	0.90	3.86E-02			
chr12.0115761694	0.74	3.86E-02			
chr18.0058082607	-0.85	3.87E-02			
chr13.0094394841	0.83	3.87E-02			
chr12.0011069368	-0.74	3.87E-02	PRR4		
chr10.0047627474	-0.94	3.87E-02	ANTXRLP1		
chr16.0056310209	0.82	3.87E-02	GNAO1	X	
chr14.0099473981	0.71	3.87E-02			
chr9.0104249370	1.28	3.88E-02	TMEM246		

chr15.0070433178	1.03	3.88E-02				
chr2.0064021225	-0.75	3.89E-02	WDPCP			
chr11.0024130715	-0.63	3.89E-02				
chr16.0024518034	0.82	3.89E-02				
chr5.0150376736	0.72	3.89E-02				
chr14.0080552094	0.89	3.89E-02				
chr3.0012698352	-1.00	3.90E-02	RAF1			
chr5.0125672242	0.91	3.90E-02				X
chr11.0123461691	0.96	3.91E-02	GRAMD1B			
chr2.0192842971	0.76	3.91E-02	AC098617.1, TMEFF2			
chr11.0070006081	0.93	3.91E-02	ANO1			
chr7.0072778744	1.05	3.91E-02				
chr13.0106635027	0.89	3.91E-02				
chr14.0088893480	0.92	3.91E-02	SPATA7			
chr5.0013802634	0.78	3.92E-02	DNAH5			
chr3.0055467569	0.72	3.92E-02				
chr16.0061703704	0.91	3.92E-02	CDH8			
chr10.0119381735	-0.76	3.92E-02				
chr12.0117066496	-0.81	3.92E-02				
chr12.0111021296	-1.30	3.93E-02				
chr11.0134399829	0.94	3.93E-02				
chr7.0142705156	0.98	3.93E-02				
chr11.0002124000	0.80	3.94E-02				
chr12.0029201938	0.85	3.95E-02				
chr12.0003140411	0.78	3.95E-02				
chr13.0041472719	-0.82	3.97E-02	TPTE2P5			
chr16.0053400387	-0.96	3.97E-02	RP11-44F14.1			
chr12.0048360477	-1.10	3.97E-02	TMEM106C			
chr2.0131288961	-0.92	3.97E-02	AC013269.4			
chr3.0029699706	-0.81	3.97E-02	RBMS3			
chr6.0137017520	-1.19	3.97E-02	MAP3K5			
chr17.0076230868	0.83	3.98E-02	TMEM235			
chr20.0062086604	-1.94	3.98E-02	KCNQ2			
chr13.0112732864	-1.20	3.98E-02				
chr11.0128294712	-1.12	3.99E-02				
chr7.0044621160	0.97	3.99E-02	TMED4			
chr16.0069889050	-0.67	4.00E-02	WWP2			
chr12.0121371899	0.88	4.01E-02				
chr5.0067769649	-0.88	4.01E-02				
chr14.0089977211	0.90	4.02E-02	RP11-33N16.3			
chr16.0014591702	-0.78	4.02E-02	PARN			

chr22.0038153720	0.85	4.02E-02	NOL12, TRIOBP		
chr20.0021709627	-0.84	4.02E-02			
chr12.0014381690	0.82	4.02E-02			
chr2.0027325377	0.84	4.03E-02	CGREF1		
chr12.0048343861	0.78	4.03E-02			
chr13.0068630715	-0.79	4.06E-02			
chr14.0105362383	-0.47	4.06E-02	CEP170B		
chr13.0113087924	-1.10	4.06E-02	SPACA7		
chr3.0167538268	-0.74	4.06E-02	SERPINI1		
chr5.0120145412	-0.78	4.06E-02			
chr15.0081041002	0.86	4.07E-02	ABHD17C		
chr10.0049703673	-0.72	4.07E-02	ARHGAP22		
chr18.0007062524	-1.03	4.08E-02	LAMA1		X
chr7.0095966645	0.79	4.08E-02			
chr3.0054985490	-1.29	4.08E-02	CACNA2D3, LRTM1		
chr18.0011887551	-0.93	4.09E-02	MPPE1		
chr20.0030667631	-0.77	4.09E-02	HCK		X
chr20.0061201229	-1.29	4.10E-02			
chr19.0015157692	0.77	4.10E-02			
chr5.0136983239	-0.72	4.10E-02	KLHL3		
chr18.0043745227	-0.80	4.11E-02			
chr14.0104486538	0.85	4.14E-02	TDRD9		
chr15.0070601410	0.91	4.14E-02			
chr20.0052476982	0.85	4.14E-02			
chr5.0057150022	0.90	4.14E-02			
chr6.0064593503	-0.76	4.14E-02			
chr2.0239051535	-0.77	4.15E-02	KLHL30		
chr10.0020897233	-0.66	4.15E-02			
chr20.0059641710	1.19	4.16E-02			X
chr18.0073920398	-0.87	4.16E-02	MBP		
chr18.0074759150	-0.93	4.16E-02			
chr7.0128476506	0.83	4.17E-02	FLNC		
chr3.0177310875	0.96	4.17E-02	LINC00578		
chr12.0052735176	0.79	4.17E-02			
chr6.0042416374	1.00	4.17E-02	TRERF1		
chr16.0008943462	0.86	4.17E-02	RP11-77H9.2		
chr15.0088742551	0.95	4.17E-02			
chr22.0017715447	-0.84	4.17E-02			
chr16.0006735523	-0.91	4.17E-02	RP11-420N3.2		
chr18.0034823936	-1.27	4.17E-02	RP11-95O2.5		
chr10.0035244295	-0.71	4.17E-02			X

chr2.0097217971	0.75	4.17E-02	ARID5A		
chr14.0100614373	-0.53	4.17E-02	DEGS2		
chr6.0129931701	0.78	4.18E-02	ARHGAP18		
chr5.0172685779	0.77	4.18E-02			
chr5.0029564329	0.97	4.19E-02			
chr14.0055604957	0.79	4.21E-02	LGALS3		
chr14.0021542358	0.75	4.21E-02	ARHGEF40		X
chr12.0120784045	0.74	4.22E-02	MSI1		
chr20.0057394000	-1.31	4.23E-02	GNAS-AS1		
chr18.0007109204	-0.79	4.23E-02			
chr19.0048481656	-0.87	4.24E-02	BSPH1		
chr22.0032939718	0.75	4.24E-02	SYN3		
chr16.0089985975	0.76	4.25E-02	MC1R, AC092143.1, TUBB3		
chr2.0002791603	-0.90	4.25E-02			
chr14.0045388379	-0.72	4.25E-02			
chr8.0143190895	-1.05	4.26E-02			
chr5.0002237179	0.78	4.26E-02			
chr6.0157152467	0.83	4.26E-02	ARID1B		
chr5.0166490935	-0.91	4.26E-02			
chr21.0011090141	0.62	4.26E-02	BAGE2		
chr6.0149937814	0.70	4.26E-02	KATNA1		
chr12.0125896092	-0.88	4.26E-02	TMEM132B		
chr5.0134374357	0.79	4.27E-02	C5orf66		
chr15.0038773631	-0.81	4.27E-02	FAM98B		
chr5.0036158752	-1.03	4.27E-02	SKP2		
chr17.0036286930	1.72	4.27E-02	TBC1D3F		
chr17.0000947293	0.82	4.27E-02			
chr11.0038706225	0.70	4.27E-02			
chr6.0056842544	0.68	4.28E-02	BEND6		
chr3.0147130343	1.16	4.28E-02	ZIC1		
chr6.0064409774	-0.67	4.28E-02	PHF3		
chr11.0019914387	0.75	4.28E-02			
chr14.0096890099	0.99	4.28E-02	AK7, PEBP1P1		
chr13.0050700606	-1.26	4.29E-02	DLEU1		
chr16.0022622146	-0.74	4.29E-02	RP11-105C19.2		
chr5.0001857029	-0.83	4.29E-02			
chr20.0004410326	0.88	4.29E-02			
chr6.0037591834	0.79	4.29E-02			
chr20.0062595495	0.84	4.30E-02	ZNF512B		
chr12.0105595125	-0.78	4.31E-02	APPL2		
chr14.0045712025	0.96	4.32E-02	MIS18BP1		

chr13.0074125683	0.92	4.32E-02	LINC00393		
chr15.0101230722	0.95	4.32E-02			
chr3.0124501859	-1.09	4.33E-02	ITGB5, ITGB5-AS1	X	
chr15.0075471349	-1.16	4.33E-02			
chr2.0107872629	0.68	4.35E-02	AC006227.1		
chr13.0059923263	0.95	4.36E-02			
chr20.0019505014	0.94	4.37E-02	SLC24A3		
chr15.0065254751	-0.83	4.38E-02			
chr14.0045511394	0.86	4.38E-02	KLHL28, FAM179B		
chr18.0036591252	0.81	4.38E-02			
chr14.0034925961	0.73	4.38E-02	EGLN3, SPTSSA		
chr2.0021265146	0.78	4.38E-02	APOB		
chr18.0003303444	0.74	4.38E-02	RP11-838N2.5		
chr6.0161034892	-1.68	4.39E-02			
chr13.0046815728	0.76	4.39E-02	LRRC63		
chr12.0108923729	0.85	4.39E-02	SART3		
chr18.0035426619	0.80	4.39E-02			
chr12.0129876394	0.77	4.39E-02			
chr10.0097063113	0.82	4.39E-02			
chr2.0045170583	0.78	4.40E-02	SIX3		
chr8.0010004530	-0.66	4.41E-02			
chr22.0032955536	-0.87	4.41E-02			
chr13.0078313090	-0.51	4.41E-02	SLAIN1		
chr12.0094075470	0.84	4.42E-02	CRADD		
chr20.0039774739	1.20	4.42E-02	PLCG1		
chr22.0045606206	0.81	4.43E-02	KIAA0930		
chr11.0077903359	-1.33	4.44E-02	USP35		
chr16.0048541221	0.72	4.45E-02			
chr6.0043196488	-0.83	4.46E-02	DNPH1		
chr11.0086628083	1.02	4.46E-02	PRSS23, RP11-736K20.5		
chr12.0006868231	0.90	4.46E-02	MLF2		
chr9.0018487650	0.78	4.46E-02	ADAMTSL1		
chr14.0020925176	-1.06	4.47E-02	APEX1		
chr15.0046178808	-0.70	4.47E-02	RP11-718O11.1		
chr5.0156893973	0.74	4.47E-02	ADAM19, NIPAL4		
chr14.0081388455	-0.83	4.48E-02	CEP128		
chr11.0002291427	-0.85	4.48E-02	ASCL2		
chr3.0138329586	-0.80	4.50E-02	FAIM		
chr16.0018418518	-1.09	4.50E-02	NPIPA8		
chr16.0059132785	0.70	4.51E-02			
chr18.0036282984	0.95	4.52E-02			

chr22.0049851087	-0.99	4.52E-02	C22orf34		
chr20.0010823170	0.97	4.52E-02	RP11-103J8.1		
chr9.0138649349	1.05	4.52E-02	KCNT1		
chr12.0027218245	0.92	4.53E-02	MED21		
chr13.0099687193	1.06	4.53E-02	DOCK9		
chr10.0006094904	0.80	4.54E-02	IL2RA		
chr18.0048045670	0.70	4.54E-02			X
chr20.0061696282	-1.34	4.54E-02	RP11-305P22.9		
chr18.0056143957	-1.22	4.54E-02			X
chr20.0000545112	1.09	4.54E-02			
chr14.0035805016	-0.76	4.54E-02			
chr6.0031116343	0.83	4.55E-02	CCHCR1		
chr12.0103285072	-0.86	4.55E-02	PAH		
chr20.0004983137	-1.36	4.55E-02	SLC23A2		
chr15.0048366878	0.95	4.55E-02			
chr5.0013843916	0.78	4.57E-02			
chr5.0071546775	-0.91	4.58E-02	MRPS27		
chr5.0179126759	1.32	4.58E-02			
chr20.0042363457	0.85	4.58E-02			
chr14.0099855064	-0.96	4.58E-02			
chr2.0005435898	-0.86	4.59E-02			
chr12.0130962090	0.89	4.59E-02			
chr12.0114887843	0.62	4.59E-02			
chr9.0046799376	0.84	4.60E-02			
chr13.0080202808	1.16	4.60E-02			
chr13.0057713776	2.36	4.61E-02			
chr4.0108519668	0.62	4.61E-02	PAPSS1		
chr12.0122523166	0.76	4.61E-02	MLXIP		
chr12.0097528239	-0.86	4.61E-02	RP11-541G9.1		
chr3.0123392817	-0.84	4.61E-02			
chr6.0150612047	1.01	4.61E-02			
chr20.0042767780	-0.44	4.62E-02	JPH2		
chr9.0133537741	1.22	4.62E-02			
chr2.0023812332	0.88	4.62E-02	KLHL29		
chr19.0046917438	1.00	4.64E-02			
chr5.0095194629	-1.11	4.64E-02	C5orf27		
chr20.0040369703	0.82	4.64E-02			
chr9.0004277896	1.20	4.64E-02	GLIS3		
chr22.0037898653	-0.79	4.65E-02	CARD10		
chr11.0070461935	0.72	4.65E-02	SHANK2		
chr9.0069655629	-0.94	4.65E-02	AL445665.1		

chr14.0104817261	-1.49	4.68E-02			
chr16.0032029419	1.03	4.68E-02	RP11-1166P10.6		
chr14.0095134050	0.96	4.68E-02			
chr3.0079397217	0.73	4.68E-02	ROBO1		
chr2.0151525923	0.66	4.68E-02			
chr9.0006651347	0.67	4.68E-02	RP11-390F4.6		
chr16.0081889624	0.88	4.68E-02	PLCG2		
chr11.0096219833	0.77	4.68E-02	JRKL, JRKL-AS1		
chr13.0026498399	0.82	4.68E-02			X
chr3.0189791239	-1.13	4.68E-02	LEPREL1		
chr6.0040954533	0.86	4.68E-02			X
chr16.0013107677	0.72	4.70E-02	SHISA9		
chr17.0079996194	0.92	4.70E-02	RP13-650J16.1		
chr6.0149094521	-0.76	4.70E-02	UST		
chr7.0111845429	0.87	4.70E-02	DOCK4		
chr14.0091071955	0.84	4.70E-02	TTC7B		
chr7.0124571125	-0.88	4.70E-02	POT1-AS1		
chr6.0165332638	0.72	4.71E-02			
chr2.0238320931	0.83	4.71E-02	COL6A3	X	
chr13.0026245179	-0.85	4.71E-02			
chr9.0126777284	0.68	4.71E-02	LHX2		
chr13.0069678204	0.90	4.72E-02			
chr16.0068734681	0.83	4.72E-02	CDH3		
chr9.0138111130	0.91	4.74E-02	RP11-399H11.3		
chr18.0056037944	-0.87	4.75E-02			
chr5.0046194285	-0.86	4.76E-02			
chr17.0019019082	-0.84	4.76E-02			
chr20.0023155959	0.85	4.76E-02			
chr20.0024664643	0.92	4.77E-02			
chr6.0135083056	0.69	4.78E-02			
chr6.0158051172	0.93	4.78E-02	ZDHHC14		
chr15.0099177743	-0.80	4.79E-02			X
chr14.0026099101	1.26	4.80E-02			
chr9.0111559847	0.75	4.80E-02			
chr13.0100174570	0.80	4.80E-02	TM9SF2		
chr13.0044948756	1.07	4.80E-02	SERP2		
chr12.0127420168	0.89	4.80E-02	RP11-575F12.1		
chr2.0223166989	0.90	4.80E-02	CCDC140		
chr20.0047082687	0.89	4.80E-02			
chr20.0036134828	-0.81	4.80E-02	BLCAP		X
chr18.0031894875	0.91	4.80E-02			

chr18.0008069950	-1.00	4.80E-02			
chr18.0073520541	-0.52	4.80E-02			
chr17.0070357736	0.78	4.80E-02	LINC00511		
chr6.0114651208	0.72	4.81E-02	RP3-399L15.3, HS3ST5		X
chr16.0020444478	1.14	4.81E-02	ACSM5		
chr21.0043893935	0.67	4.81E-02	RSPH1		
chr11.0017593750	-0.91	4.81E-02	OTOG		
chr18.0068910726	1.21	4.81E-02			
chr2.0143885175	0.74	4.82E-02	ARHGAP15		
chr12.0054441154	1.04	4.83E-02			X
chr18.0022272403	0.81	4.84E-02			
chr18.0006837701	0.64	4.84E-02	ARHGAP28		
chr12.0050507247	-0.82	4.84E-02	COX14, RP4-605O3.4		
chr3.0181431204	-1.07	4.84E-02	SOX2-OT, SOX2		
chr8.0117644009	0.81	4.85E-02			
chr20.0052475838	0.83	4.85E-02			
chr13.0027414220	1.41	4.85E-02			
chr6.0167422116	-0.99	4.85E-02	FGFR1OP, RP11-517H2.6		
chr10.0071266674	0.68	4.85E-02	TSPAN15		
chr16.0089927438	-0.97	4.86E-02	SPIRE2		
chr6.0169494777	0.87	4.86E-02			
chr17.0041656132	1.03	4.86E-02	RP11-392O1.4, ETV4		
chr18.0077339715	0.83	4.86E-02	RP11-567M16.1		
chr14.0093191004	-0.53	4.86E-02	LGMN		
chr2.0065538907	0.72	4.87E-02	SPRED2		
chr2.0134057040	0.65	4.87E-02	NCKAP5		
chr20.0045710639	-0.84	4.87E-02	EYA2		
chr11.0011488647	-0.68	4.87E-02	GALNT18		
chr15.0029504631	0.93	4.87E-02	FAM189A1		
chr15.0021059321	-1.52	4.88E-02	POTEB2		
chr5.0005255542	0.74	4.91E-02	ADAMTS16		
chr5.0002831599	0.85	4.91E-02	RP11-468D11.1		
chr5.0001005654	-0.99	4.91E-02	RP11-43F13.4		
chr6.0111172259	-0.72	4.91E-02			
chr14.0073127258	-0.84	4.91E-02	DPF3		
chr19.0022857817	0.72	4.92E-02	CTC-457E21.9		
chr18.0049179172	-1.20	4.92E-02			
chr6.0051719910	-0.97	4.92E-02	PKHD1		X
chr5.0003988100	0.94	4.92E-02			
chr16.0003696466	0.97	4.92E-02	DNASE1		
chr3.0039328066	0.71	4.92E-02	RP11-331G2.6		

chr12.0130083075	0.95	4.92E-02			
chr16.0059568501	0.70	4.92E-02			
chr18.0032397156	0.78	4.94E-02	DTNA	X	X
chr20.0005006235	-0.88	4.94E-02			
chr2.0150195221	0.75	4.95E-02	LYPD6		
chr18.0049041058	0.81	4.95E-02			
chr17.0001057768	0.93	4.95E-02			
chr12.0062497446	0.75	4.96E-02			
chr12.0002505095	0.82	4.96E-02	CACNA1C	X	
chr18.0044221958	-0.53	4.96E-02	LOXHD1		
chr20.0017996650	-0.71	4.97E-02	OVOL2		
chr6.0115910955	0.83	4.97E-02			
chr5.0174832325	0.85	4.97E-02			
chr3.0189389138	0.67	4.97E-02	TP63		
chr16.0071132400	0.78	4.97E-02	HYDIN		
chr11.0072553556	0.84	4.98E-02	ATG16L2, FCHSD2		
chr11.0120962446	-0.78	4.99E-02			
